# Evaluating the impact of community health worker certification in Massachusetts: Design, methods, and anticipated results of the Massachusetts community health worker workforce survey

**DOI:** 10.3389/fpubh.2022.1043668

**Published:** 2023-01-12

**Authors:** Victoria M. Nielsen, W. W. Sanouri Ursprung, Glory Song, Gail Hirsch, Theresa Mason, Claire Santarelli, Erica Guimaraes, Erica Marshall, Caitlin G. Allen, Pei-Pei Lei, Diane Brown, Bittie Behl-Chadha

**Affiliations:** ^1^Massachusetts Department of Public Health, Office of Statistics and Evaluation, Bureau of Community Health and Prevention, Boston, MA, United States; ^2^Massachusetts Department of Public Health, Office of Community Health Workers, Bureau of Community Health and Prevention, Boston, MA, United States; ^3^Division of Health Protection and Promotion, Massachusetts Department of Public Health, Bureau of Community Health and Prevention, Boston, MA, United States; ^4^Mass General Brigham, Boston, MA, United States; ^5^Division of Community-Based Prevention and Care, Massachusetts Department of Public Health, Bureau of Community Health and Prevention, Boston, MA, United States; ^6^Department of Public Health Sciences, College of Medicine, Medical University of South Carolina, Charleston, SC, United States; ^7^Office of Survey Research, University of Massachusetts Chan Medical School, Shrewsbury, MA, United States

**Keywords:** community health workers, surveys and questionnaires, certification, methods, healthcare reform

## Abstract

**Background:**

Professional certification of community health workers (CHWs) is a debated topic. Although intended to promote CHWs, certification may have unintended impacts given the grassroots nature of the workforce. As such, both intended effects and unintended adverse effects should be carefully evaluated. However, there is a lack of published literature describing such effective evaluations with a robust methodology. In this methods paper, we describe a key component of evaluating CHW certification in Massachusetts-the Massachusetts CHW Workforce Survey.

**Methods:**

Design of the surveys was informed by a program theory framework that delineated both positive and negative potential impacts of Massachusetts CHW certification on CHWs and CHW employers. Using this framework, we developed measures of interest and preliminary CHW and CHW employer surveys. To validate and refine the surveys, we conducted cognitive interviews with CHWs and CHW employers. We then finalized survey tools with input from state and national stakeholders, CHWs, and CHW employers. Our sample consisted of three frames based on where CHWs are most likely to be employed in Massachusetts: acute care hospitals, community-based organizations, and ambulatory care health centers, primarily community health centers and federally qualified health centers. We then undertook extensive outreach efforts to determine whether each organization employed CHWs and to obtain CHW and CHW employer contact information. Our statistical analysis of the data utilized inverse probability score weighting accounting for organizational, site, and individual response.

**Anticipated results:**

Wave one of the survey was administered in 2016 prior to launch of Massachusetts CHW certification and wave two in 2021. We report descriptive statistics of the three sample frames and response rates of each survey for each wave. Further, we describe select anticipated results related to certification, including outcomes of the program theory framework.

**Conclusions:**

The Massachusetts CHW Workforce Survey is the culmination of 5 years of effort to evaluate the impact of CHW certification in Massachusetts. Our comprehensive description of our methodology addresses an important gap in CHW research literature. The rigorous design, administration, and analysis of our surveys ensure our findings are robust, valid, and replicable, which can be leveraged by others evaluating the CHW workforce.

## Introduction

A community health worker (CHW) is a public health worker who utilizes their unique understanding of the populations they serve to carry out several roles, such as health education, outreach, and care coordination (1). Known for shared personal experiences with the populations they serve and intimate knowledge of the communities in which they live and operate, CHWs are a critical asset in a multitude of public health activities. CHWs are known by a variety of job titles, an indication of the diverse populations with which they work and the broad range of services they provide, which often arise organically from community needs (1–3). The CHW's ability to establish trust and rapport, embedment in social networks, and thorough knowledge of the strength, resilience, and resources in the community are attributes that cannot be replicated in any other profession.

Although early research of the efficacy of CHWs was hindered by substantial methodological limitations and program implementation problems, a multitude of recent studies using robust methods has demonstrated the impact CHWs have on an array of measures and outcomes (4–7). For example, a systematic review of CHW-led interventions in populations with pediatric asthma suggests that such interventions can reduce symptoms, decrease activity limitations, and reduce emergency and urgent care use (6). Another systematic review of CHW interventions among adult populations with diabetes suggests that CHWs have significant impacts on physical health, knowledge of diabetes, self-care, and wellbeing (7). Although further research is needed, including more rigorous integration of theoretical frameworks into program design and evaluation, the literature increasingly demonstrates the role CHWs play in improving health outcomes of underserved populations (4, 6). Further, the passage of the Patient Protection and Affordable Care Act in 2010 not only increased healthcare's accountability for mitigating upstream factors that can be addressed by CHWs, but also facilitated CHW integration into healthcare teams and delivery models (8–12). Taken together, the legitimate role CHWs play in improving health in both community and healthcare settings has become apparent to a wider audience in the past two decades.

Given this wider recognition, professional certification of CHWs has been raised as a means to legitimize their role, produce a clearer definition of scope, and increase opportunities for sustainable financing and reimbursement (13, 14). However, there is no national certification of CHWs and states pursuing certification have taken heterogeneous approaches. In 2010, the Massachusetts legislature passed an Act Establishing a Board of Certification of Community Health Workers, which in part began the creation of a process of certifying Massachusetts CHWs (15). Over the next decade, CHWs, CHW employers, state officials, and advocates worked to implement an equitable, accessible certification process. In 2018, CHW certification by the experience pathway was launched, which is administered by the Massachusetts Department of Public Health (MDPH) Bureau of Health Professions Licensure (16). The combined training and work experience certification pathway launched in 2021 and at the time of this publication there are four board-approved core competency training and education programs (17).

Although certification is intended to champion and promote the CHW workforce, there may be unintentional adverse impacts (13, 18). One element that makes CHWs so effective is their similarity with the populations they serve, a characteristic that certification may alter (13). Shifts in the demographics of the CHW workforce, loss of job opportunities to CHWs who are not certified, inequitable increases in salary by demographics or certification status, and the cost and burden of obtaining certification are other notable concerns (13, 14, 18). Finally, certification must always be considered voluntary and should not be utilized as a requirement for employment or promotion. As such, CHW employers' certification-related perceptions and requirements should also be monitored.

Population surveys of CHWs and CHW employers are an important tool in evaluating the impact of certification; however there is a notable gap in the published literature describing such methods. Additionally, conducting population surveys presents several methodological challenges (19). Previously published surveys and evaluations of the impact of CHW certification frequently do not perform statistical adjustment of the data; rely on non-probability sampling; do not collect data on important confounders, such as type of organization in which the CHW is employed; or have limited generalizability due to an unclear definition of the CHWs included in the sample, especially given the variety of titles under which CHWs operate (20–26). Given the far-reaching implications such evaluations may have, it is essential that methodology be robust to ensure results are valid and generalizable. In this manuscript we describe our rigorous and replicable methodology in the design, administration, and statistical analysis of the Massachusetts CHW Workforce Survey, which is a key tool in evaluating the launch of Massachusetts CHW certification.

## Materials and equipment

### Institutional review board

Survey procedures were reviewed by the University of Massachusetts Chan Medical School (UMass Chan) institutional review board. The project was deemed non-human subjects research. Additionally, responses and contact information are considered confidential information and are maintained behind MDPH and UMass Chan Office of Survey Research firewalls on password protected computers. Any release of aggregate survey data must adhere to standards set by the MDPH Privacy and Data Compliance Office (PDCO).

### Sample frame development

We began development of the survey sample by searching for a comprehensive list of all organizations in the state within each of the following core frames: acute care hospitals (hospital frame); community-based organizations (CBO frame); and Massachusetts community health centers (CHCs) and federally qualified health centers (FQHCs) (health center frame). These three core frames do not reflect an exhaustive list of settings in which CHWs are employed; rather, they reflect evaluation priorities in Massachusetts as well as findings from past surveys conducted in Massachusetts that indicate where CHWs are likely to be employed (22). Additional frames, such as mental health centers and outpatient pediatric clinics, were considered but could not be included due to resource limitations. However, because CHCs and FQHCs provide mental health and pediatric care, our survey likely did reach a sample of CHWs providing these services.

We created preliminary lists of organizations with contact information in each frame using publicly available sources: a full list of acute care hospitals and phone numbers was collated from the MDPH Bureau of Healthcare Safety and Quality, the state government entity responsible for licensing of all healthcare providers in the state; a list of CHCs and FQHCs were obtained from the Massachusetts League of Community Health Centers and Health Resources and Services Administration, respectively; and CBOs were obtained *via* existing state public health records, exhaustive internet search, historical knowledge of CBOs in Massachusetts from a network of key informants, and organization registries kept by the Massachusetts Association of Community Health Workers (MACHW) (20, 27). Unlike the former two sample frames, identification of CBOs was challenging as there is no comprehensive list of such organizations; nonetheless, we undertook extensive efforts to include as many CBOs as possible. Examples of CBOs included in our sample frame include organizations providing addiction recovery services; access to housing and food; faith-based organizations; and domestic violence services. We then undertook an exhaustive internet search to determine whether organizations in each frame operated at multiple sites in Massachusetts and to obtain publicly available contact information for each site.

The three core frames are comprised of the following: 43 hospitals in wave one and 42 in wave two, 56 health centers in wave one and 57 in wave two, and 158 CBOs in wave one and 171 in wave two. Unfortunately, expanding to additional frames in the same comprehensive and systematic fashion where CHWs are employed, such as additional community health clinics that are not CHCs or FQHCs, hospitals focusing on special populations, and state and local health departments, was not feasible given time and funding limitations. As such, we incorporated into each frame a small selection of additional organizations that serve similar functions as their assigned frame: urgent care, healthcare networks, pediatric hospitals, and veteran's hospitals in the hospital frame (*n* = 7 in wave one, *n* = 8 in wave two); community-based health clinics focusing on specialty services such as women's health and reproductive health in the health center frame (*n* = 10 in each wave); and divisions of health departments providing community-based services in the CBO frame (*n* = 10 in each wave). Although these are not comprehensive, we felt it was nonetheless important to include these additional organizations to reach as many CHWs as possible in Massachusetts.

### Survey tool development

Survey tools were developed in collaboration with the MDPH Office of CHWs and Office of Statistics and Evaluation; the Board of Certification of CHWs; MACHW; and the UMass Chan Office of Survey Research. Design of the surveys was guided by our development of a program theory framework that broadly endeavored to capture how launch of Massachusetts CHW certification could both positively and negatively impact the Massachusetts CHW workforce. This framework was developed from two sources. First, we conducted an extensive review of the literature, including peer reviewed articles, gray literature, and reports, that identified gaps in CHW certification research and evaluation, perceptions and concerns regarding CHW certification, previous surveys of the CHW workforce and CHW employers, and findings from other states that had launched CHW certification. The second source was extensive engagement with state and national veteran CHW subject matter experts (SME). This includes experts with decades of expertise in CHW-related research (GH, TM); perspectives gathered from engaging with CHWs and CHW employers across Massachusetts; and input from local, state, and national partners. These efforts resulted in the derivation of several key evaluation questions. However, due to resource limitations, five of these were prioritized to assess the impact of CHW certification in Massachusetts. Using these evaluation questions as a guide and leveraging the expertise of researchers with decades of experience in survey research, design, psychometrics, and administration (WWSU, CA, TM, BBC, PPL, DB), we developed measures and corresponding CHW and CHW employer survey questions.

Supplement A contains a matrix of these evaluation questions, measures, and corresponding CHW and CHW survey questions. We aligned select questions on the CHW and CHW employer surveys to enable contrasts between CHW employer and CHW perspectives, which are aligned in the table text. With these measures defined, we then designed activities that would promote Massachusetts CHW certification as well as support all CHWs in Massachusetts regardless of decision to seek certification, such as: increasing awareness of the Massachusetts CHW Board of Certification; sharing training opportunities offering continuing education unit (CEU) credits with CHWs; establishing core competency training programs and centers by the Massachusetts CHW Board of Certification; and providing targeted technical assistance to CHWs and CHW employers. Examples of technical assistance include processes to integrate CHWs into clinical care teams and how to advocate for CHW access to and documentation of their work in the electronic health record.

### Survey tool refinement and validation

Once we developed the preliminary CHW and CHW employer surveys, the UMass Chan Office of Survey Research conducted cognitive interviews with 10 CHWs and 10 CHW employers during which they reviewed survey questions and responses as well as obtained additional information to inform survey design. Interview participants spanned the three organization types and each interview was ~45–50 min in length. Verbal consent was obtained, and participants were notified that they could discontinue at any time.

In addition to reviewing the survey tools, the cognitive interviews gathered additional information regarding employment attributes, funding sources, roles and activities, integration into care teams, perceived value of CHW work within the organization, training, and perceived benefits and barriers related to certification. See Supplements B, C for the CHW and CHW employer interview guides, respectively. The cognitive interviews served four purposes: provided feedback on the CHW and CHW employer surveys; informed the critical topics to be included in the survey to keep survey length reasonable; assisted in the wording of the questions and response options in a way that would validly resonate with the respondents; and guided in the design of specific response options to be included with each question.

After integrating the findings of the cognitive interviews into the surveys, we conducted additional cognitive interviews with state and national CHW SMEs to further refine the clarity, appropriateness, and comprehensiveness of survey questions. Additionally, MACHW and CHW Core Consensus Project (C3) reviewed each tool in its entirety and provided feedback. Finally, the CHW survey tool and the CHW employer survey tool were reviewed by a small group of CHWs and CHW employers, several of whom brought both the CHW and CHW employer perspective. The tools were further modified based on this feedback. Although formal testing is ideal in survey development, resource limitations and funding restrictions were a challenge in conducting additional validation. However, leveraging the subject matter expertise of those developing and reviewing the survey, integration of findings from the cognitive interviews, and the tools undergoing review by several members of the target population enhanced the validity and reliability of the surveys.

As a result of these efforts, we finalized the survey tools that broadly covered the following domains: (1) Aspects of CHW employment, such as job title, pay, full or part-time employment, and organization information; (2) Role of the CHW within the organization, such as health issues addressed, health promotion activities, linkages to community resources, and work with special populations; (3) Trainings and certification of the CHW, such as receipt of the 80-h core competency training and trainings in specific disease areas; (4) Clinical care team integration, including use of electronic health records and interactions with the care team (asked of clinical organizations only); and (5) Certification, such as certification status, perceptions of certification, and ease of certification process. In the 2016 wave, the CHW survey tool was available in English only. In the 2021 wave, the CHW survey tool was translated into Spanish and Portuguese, given that these are the second and third most commonly spoken languages in Massachusetts and were the most frequently reported second languages on wave one of the CHW survey (28). Translation of the survey was intended to accommodate CHWs who were more comfortable communicating in a non-English language. The translated Spanish and Portuguese surveys were reviewed by a native Spanish and a native Portuguese speaker, respectively, to ensure accuracy and appropriateness of translations.

The English CHW employer and CHW survey tools can be found in Supplements D, E, respectively. In the attached supplements, questions that align between the CHW and CHW employer surveys are indicated in brackets (blue font) on the CHW survey tool. We then built the survey tools into the online survey platform Confirmit.

## Methods

### Obtaining contact information and survey administration

After collecting publicly available contact information for all organizations and sites within the sample frames, we initiated outreach prior to each wave of the survey launch to ascertain whether the organization and affiliated sites employed CHWs and to obtain the email addresses of CHW employers and CHWs working at the organization. MDPH and UMass Chan interviewing staff contacted each organization in the sample frame using a call script. Given the variety of roles and job titles CHWs have, interviewers used a fact sheet that provided detailed information on the roles, job titles, and responsibilities of CHWs (Supplement F). If during this initial outreach the contact expressed hesitancy to provide this information, interviewers offered to email an official letter from MDPH to establish the legitimacy of the survey. Regarding the hospital frame, determining which specific department in which a CHW worked within a hospital was notably challenging. As such, while collecting contact information we made the decision to obtain information on all CHWs from the general contact number from the hospital even if we could not identify the specific department that those CHWs were affiliated with. Organizations and sites not employing CHWs were indicated as such and were deemed not eligible for the survey. Please see Table 1 for organizational characteristics of CHWs and CHW employers who provided contact information.

**Table 1:** Organizational characteristics of CHWs and CHW employers included in the sample frame.

**Organizational characteristics of the sample frame**	**Wave 1**				**Wave 2**			
	**Employers**		**CHWs**		**Employers**		**CHWs**	
	*n* ^^*^^	%^^*^^	*n* ^^*^^	%^^*^^	*n* ^^*^^	%^^*^^	*n* ^^*^^	%^^*^^
	298		871		283		948	
**Organization type**							
Hospitals	84	28.1	173	19.8	44	15.5	108	11.3
Health centers	88	29.5	254	29.1	109	38.5	336	35.4
Community-based organizations	126	42.2	444	50.9	130	45.9	504	53.1
**Organization size (based on number of employees)**							
Small (1–49)	53	17.7	120	13.7	42	14.8	182	19.1
Medium (50–500)	132	44.2	478	54.8	93	32.8	376	39.6
Large (501+)	113	37.9	273	31.3	148	52.2	390	41.1
**Neighborhood Stress Score (NSS) deciles** ^**^							
1–4 (−1.714 to −0.681)	20	6.7	106	12.1	19	6.7	62	6.5
5–7 (−0.680 to −0.228)	24	8.0	47	5.3	18	6.3	47	4.9
8 (−0.227 to 0.180)	35	11.7	92	10.5	41	14.4	156	16.4
9 (0.181 to 1.024)	56	18.7	106	12.1	50	17.6	155	16.3
10 (1.025 to 4.841)	163	54.6	520	59.7	155	54.7	528	55.6

* Column totals may not sum due to truncation and/ or missing values.

** NSS decile ranges based on NSS values for the state of Massachusetts. Select deciles were collapsed due to small cell size to protect confidentiality in adherence with the Massachusetts Department of Public Health Privacy and Data Compliance Office confidentiality procedures.

There were several important lessons learned during this process. Using fact sheets and definition of CHWs was critical since most contacts did not know what a CHW was. Receptionists at the organization's general number often were unable to provide any information; as such, interviewers found it best to ask for managers responsible for hiring and payroll. Finally, contacting human resources departments, especially in large organizations, often did not prove fruitful. Overall, interviewers found it was best to be flexible and patient, and to talk to anyone who could provide any information. Often it took multiple efforts to obtain CHW and CHW employer contact information at a given organization.

One day prior to launch of the survey, the MDPH Office of CHWs sent an official prenotification email to all CHW employers and CHWs in the sample frame notifying them of the upcoming survey. This step was intended to reinforce legitimacy of the survey and improve response rates when organizations were later contacted directly. Attached to the email was an official letter from the MDPH Office of CHWs. The survey was then administered *via* email with a link to the survey. Wave one was administered between June 14 to August 1, 2016, and wave two between April 13 to July 1, 2021.

### Statistical analysis

Once survey administration was complete, we reviewed data files for completeness, missingness, and duplication. After completion of this step, we used inverse probability score weighting (IPW) to account for non-response and permit valid population-level comparisons across survey waves. Regardless of the evaluation or research question assessed with these data (including evaluating the impact of CHW certification), IPW is a critical step in addressing bias introduced by non-response and differential response within and between waves of the survey. We generated inverse probability weights using propensity score models with PROC LOGISTIC in SAS 9.4 using a logit link function and Fisher's Scoring method (29). We ran three separate propensity score models to generate three separate sets of weights to account for response probability at different levels: organizational probability of response to initial outreach regarding organizational employment of CHWs, which included both eligible and ineligible organizations (ineligible meaning that the organization indicated that it did not employ CHWs); site probability of response to initial outreach regarding site employment of CHWs, which included both eligible and ineligible *sites* (*organizations* that confirmed that they did not employ CHWs as a whole were removed from this step); and individual CHW and CHW employer probability of response to the survey.

All three propensity score models included as predictors organization type (CBO, hospital, health center) and organization size. We determined organization size based on publicly available tax documents from ProPublica, from which we obtained the number of staff employed by the organization, excluding volunteers (30). We determined that this was a feasible approach given that the majority of the organizations in our sample frame were non-profit or not-for-profit. For the 2016 survey wave, 2015 tax filings were used and for the 2021 survey wave, 2020 tax filings were used. If tax filings were not available, filings were incomplete, or the organization was for-profit, we conducted an internet search on the organization's website to obtain these data. Employee size was categorized as small (<50 employees), medium (50–500 employees), and large (more than 500 employees).

In addition to organization type and size, the models for the site level and CHW and CHW employer level propensity scores included a Neighborhood Stress Score (NSS) variable developed by Ash et al. (31). This composite measure is derived from the American Community Survey (ACS) data of census block group estimates of poverty, education, access to transportation, and employment. A higher score indicates higher levels of neighborhood stress. We mapped NSS scores at the site level given that many organizations operate at multiple locations, which will likely vary in community characteristics. We categorized NSS into deciles, the ranges for which were determined using statewide NSS. We included NSS as a discrete variable in the models. Our inclusion of organization size, type, and NSS in the IPW endeavored to capture both organizational and community characteristics that we hypothesized to be associated with probability of response. The weights generated by each of the three stages were then multiplied together, resulting in a single weight for each respondent to the CHW and CHW employer surveys.

## Anticipated results

In wave one, the response rate was 67% for the employer survey and 63% for the CHW survey. In wave two, the response rate was 62% for the employer survey and 53% for the CHW survey. Organizational characteristics of CHW and CHW employers responding to the survey are included in Table 2. CHWs responding to each wave of our survey are primarily female, most have some college education or higher, and the majority work full time. The representation of Black or African American CHWs declined between wave one and wave two, with the reduction distributed across White and Hispanic CHWs. In wave two, 26% of CHW employers reported employing certified CHWs and 15.6% of CHWs reported being certified by the Massachusetts Board of Certification. Please see Table 3 for characteristics of CHWs by survey wave.

**Table 2:** Organizational characteristics of CHWs and CHW employers responding to the survey.

**Organizational characteristics of survey respondents**	**Wave 1**				**Wave 2**			
	**Employers**		**CHWs**		**Employers**		**CHWs**	
	*n* ^^*^^	%^^*^^	*n* ^^*^^	%^^*^^	*n* ^^*^^	%^^*^^	*n* ^^*^^	%^^*^^
	187		531		172		486	
**Organization type**								
Hospitals	48	25.6	108	20.3	33	19.1	70	14.4
Health centers	59	31.5	191	35.9	60	34.8	178	36.6
Community-based organizations	80	42.7	232	43.6	79	45.9	238	48.9
**Organization size (based on number of employees)**								
Small (1–49)	34	18.1	88	16.5	24	13.9	94	19.3
Medium (50–500)	79	42.2	259	48.7	60	34.8	200	41.1
Large (501+)	74	39.5	184	34.6	88	51.1	192	39.5
**Neighborhood Stress Score (NSS) deciles** ^**^								
1–4 (−1.714 to −0.681)	12	6.4	41	7.7	13	7.5	31	6.3
5–7 (−0.680 to −0.228)	18	9.6	35	6.5	14	8.1	29	5.9
8 (−0.227 to 0.180)	26	13.9	63	11.8	27	15.6	86	17.6
9 (0.181 to 1.024)	36	19.2	64	12.0	33	19.1	80	16.4
10 (1.025 to 4.841)	95	50.8	328	61.7	85	49.4	260	53.4

* Column totals may not sum due to truncation and/ or missing values.

** NSS decile ranges based on NSS values for the state of Massachusetts. Select deciles were collapsed due to small cell size to protect confidentiality in adherence with the Massachusetts Department of Public Health Privacy and Data Compliance Office confidentiality procedures.

**Table 3:** Characteristics of CHW respondents by survey wave.

**Demographics of responding CHWs**	**Wave 1 (2016, *n* = 531)**	**Wave 2 (2021, *n* = 486)**
	**%** ^*^	**%** ^*^
**Employment** ^**^		
Full time, paid (at least 30 h per week)	90.0	85.8
Part time, paid (<30 h per week)	10.0	13.7
Is certified^***^	NA	15.6
**Age (years)**		
18–34	39.7	26.5
35–44	23.4	26.6
45 or older	36.9	46.9
**Total years working as a CHW**		
Up to 2 years	39.7	31.1
3–10 years	38.2	43.5
11 or more years	22.1	25.3
**Gender** ^**^		
Female	80.8	78.5
Male	19.1	18.6
**Education**		
Up to some college or 2-year degree	40.3	40.5
4-year college graduate	37.5	34.6
More than 4-year college degree	22.2	24.9
**Race/ethnicity**		
Asian (non-Hispanic)	5.0	5.1
Black or African American (non-Hispanic)	19.7	13.3
Hispanic	28.1	31.9
Other (non-Hispanic)	4.9	6.2
White (non-Hispanic)	42.3	43.5
**Languages fluent enough to communicate with**		
**individuals they serve** ^****^		
Spanish	31.4	34.2
Portuguese	8.4	7.3
Haitian Creole	4.4	4.3

* Column totals may not sum due to truncation and/ or missing values. Percentages in this chart reflect weighted survey data and are variably based on the total number of CHWs who responded to the question.

** Select response options (e.g., transgender, volunteer unpaid) were collapsed due to small cell size to protect confidentiality in adherence with the Massachusetts Department of Public Health Privacy and Data Compliance Office confidentiality procedures.

*** Not applicable in wave one as Massachusetts had not launched certification.

**** Top three languages, excluding English.

Key findings will focus on the evaluation questions related to impact of certification, outlined in Supplement A. Additionally, strata will include CHWs vs. CHW employers; wave one vs. wave two; certified CHWs vs. non-certified CHWs; CHW employers reporting requiring certification for hiring vs. those reporting not requiring certification for hiring; and CHW employers reporting employing certified CHWs vs. CHW employers reporting that they do not. Note that the last three strata are available in wave two only, as Massachusetts certification launched after wave one was administered. Finally, findings may be stratified by additional variables to mitigate confounding, such as stratifying by employer characteristics.

An exhaustive list of anticipated results is beyond the scope of this paper. Examples of anticipated findings include whether employers are leveraging stable funding sources for CHW positions; if there are shifts in the perceived value of CHWs and CHW certification; whether there are changes in trainings and promotion opportunities available to CHWs; and whether CHWs have become better integrated into care teams. Additionally, we will assess awareness of Massachusetts CHW certification and ease of and attitudes toward obtaining certification in Massachusetts. Finally, we will further analyze shifts in the CHW workforce between survey wave one and wave two. CHW demographics we will analyze include gender, educational attainment, number of years working as a CHW, income, age, race, and ethnicity. However, if any changes are detected, they are likely multifactorial and not necessarily the result of launch of certification, such as natural variation in the workforce and impacts of the Coronavirus Disease-19 (COVID-19) pandemic. Differentiating these factors will become more feasible with additional administrations of the surveys.

## Discussion

Using a rigorous, systematic, and replicable approach, the Massachusetts CHW Workforce Survey serves as a critical tool to monitor the impact of CHW certification in Massachusetts and describe a large, growing, and diverse workforce. In conjunction with ongoing outreach and evaluation efforts conducted by the MDPH Office of CHWs and their partners, findings from these surveys will be critical in assessing impacts of certification in Massachusetts. The rigorous and methodological documentation of our survey methods increases feasibility of administering additional waves of the survey to monitor change over time and replicability of findings. Additionally, researchers, governments, and CHW advocates looking to administer their own surveys can utilize our tools and approaches to inform their work in conjunction with tools and resources currently available through the National Association of Community Health workers (NACHW), the Centers for Disease Control and Prevention, and other organizations (14, 32). Given the gap in the literature describing rigorous evaluations of CHW certification, it is not surprising that over the 6 years since we designed and launched the surveys we received numerous requests regarding best practices in evaluating CHW certification-including our survey tools and methods-from local, national, and international CHW advocates, policymakers, and researchers. This manuscript describing our methods addresses this pressing need in the CHW research community.

The Massachusetts CHW Workforce Survey is not the first large-scale assessment of CHWs and their employers in Massachusetts. In the early 2000s, MDPH administered CHW and CHW employer surveys. Significant outreach efforts prior to the administration of the survey identified 806 CHWs and 155 employers (21). Additionally, Section 110 of Chapter 58 of the Acts of 2006 (Massachusetts Healthcare Reform) directed MDPH to conduct a workforce assessment and develop recommendations for a CHW program (33). In 2008, the resulting survey identified 2,932 CHWs across the state (22). In addition to establishing a demographic profile of Massachusetts CHWs, these surveys gathered critical data on roles, opportunities, and barriers facing the CHW workforce that not only drove state priorities but informed our survey design and methods as well (21, 22). Further, the 2008 survey finding that CHWs are most likely to work in CHCs, hospitals, and CBOs informed the development of our sample frame (22).

There have also been national efforts to describe and identify needs and changes in the CHW workforce as well as evaluate potential impacts of certification in the United States. In 2007, the Health Resources and Services Administration (HRSA) of the United States Department of Health and Human Services released a report based on findings of the CHW National Workforce Study (34). This project included a survey of CHW employers in all 50 states and the District of Columbia, which was supplemented with interviews conducted with employers and CHWs in 4 states. This research effort returned a wealth of data, including estimates of the size and demographics of the CHW workforce nationally and in each state (including 2,441 in Massachusetts); CHW income; populations served and roles within their organizations; education and training; and employer characteristics. In addition to the survey and interview findings, this report also exhaustively reviewed CHW certification programs in the United States and outlined requirements, fees, and CHW perceptions of certification (34). Since this landmark report, multiple state and national surveys have also assessed various aspects of the CHW workforce and CHW employers (3, 23–26, 35). Further, a national evaluation of the impact of state-level CHW certification and Medicaid reimbursement on CHW wages and turnover returned mixed results, and it is still unclear how state policies impact CHW employment (25, 36). Nevertheless, this study addressed a critical question related to CHW certification, including potential inequitable impacts by CHW subpopulation (25).

Our research adds to these surveys and evaluations by providing not only survey questionnaires, but other tools we developed as well as detailed information on the development of our sample frames. Further, our methods are to the best of our knowledge one of the few focused primarily on evaluation of state CHW certification. Regarding the sample frames, systematic and replicable construction of the sample frame is critical to conceptualize and generalize the results of the survey and increase the validity of conclusions drawn between survey waves, unlike non-probability sampling frequently employed by other surveys. Further, unclear definitions of the sample frame in previous studies impact generalizability of findings. Collection of data beyond basic CHW demographics is critical to validly evaluate impact of certification-such as organizational characteristics where CHWs are employed and length of time a CHW has worked. Failure to do so can result in significant confounding and bias study conclusions. In contrast, our well-defined sample frame along with extensive individual and organizational data permits more robust interpretation, control of confounding, and conceptualization of findings, which is especially critical given the diverse settings, job titles, and functions of CHWs.

In addition, our implementation of IPW accounts for non-response and differential response within and between survey waves. IPW is a well-documented method of reducing bias in survey research and is essential for drawing robust conclusions from findings compared to unweighted frequencies and percentages often utilized by other surveys. Although no survey analysis method can eliminate bias, our accounting for organizational and community characteristics potentially associated with response probability lessens risk of bias in our findings. The entirety of the process, from survey design, survey administration, construction of sample frames, and statistical analysis (including weighting variables) are replicable in other states and can be leveraged by researchers undertaking evaluations of the CHW workforce. For example, the variables we included in the IPW (e.g., census data, tax documents) and used to construct the sample frame (e.g., UDS HRSA data, list of all acute care hospitals in the state) are largely publicly available. Finally, the forthcoming analysis of our survey results will add to the literature by providing critical information on several important topics related to CHW certification, such as potential adverse and inequitable impacts of certification, CHW integration into healthcare teams, and shifts in CHW employment, including job opportunities, funding, salary, and training.

Our development and administration of the survey resulted in important lessons learned that may be useful for organizations interested in undertaking similar efforts. What cannot be understated is the role CHWs, CHW SMEs, advocacy organizations, and networks played in the development and administration of the survey. Designing valid questions that align with the most pressing needs CHWs face, including potential positive and negative impacts of certification, was crucial to ensure that the results are meaningful and actionable. The qualitative findings were an important component of this, as they aided us in identifying additional outcomes of interest from both the CHW and CHW employer perspectives. Additionally, leveraging networks to ensure as many CHWs are reached as possible and increasing response rates was paramount. Further, applying rigorous, statistical analysis of the data, including accounting for confounding and differential responses, is essential to draw valid, population conclusions within and between waves of the survey. Albeit no survey is without limitations, the extensive analytical efforts we undertook facilitate findings that are robust and replicable.

There are important limitations of this survey to note. While this survey reached a large sample of CHWs and CHW employers in Massachusetts, previous surveys suggest that the number of CHWs in Massachusetts is higher (21, 22, 34). However, this is not unexpected, as we limited the survey to select settings due to limited funding availability and evaluation priorities. As such, our findings will not be generalizable beyond the selected sample frames. Further, all three sample frames are fluid, given the constantly shifting nature of the healthcare and CBO landscape in Massachusetts. The survey only being available electronically may have impacted participation among CHWs and CHW employers with limited internet access or without reliable access to a computer or smart device. Although translation of the survey into Spanish and Portuguese likely permitted us to reach a larger sample of CHWs, some CHWs may have been unable to complete the survey due to limited translations available. Additionally, the first wave of the survey was available only in English, which may impact interpretation between the two survey waves. However, in wave two only a small number of translated surveys were completed (25 of 486 completed surveys). Given this, we feel this likely would not impact interpretation of findings between wave one and wave two. The timeline for wave two of the survey was significantly impacted by the COVID-19 pandemic, and collection of contact information was challenging due to increased telework. Verifying the accuracy of CHWs and CHW employers included in the survey is not feasible. This may have resulted in the inclusion of non-CHWs in the sample; however, our use of fact sheets and call scripts by interviewers while collecting contact information likely minimized this. Our use of a cross-sectional design-albeit repeated and capturing workforce information pre- and post-certification launch-limits causal inference and caution must be taken to not overstate findings. Finally, the CHW Workforce Survey is just one tool to describe the CHW workforce in Massachusetts and monitor the impacts of certification. The MDPH Office of CHWs and partners undertake ongoing evaluation and research efforts that are equally essential, including conducting outreach, focus groups, and key informant interviews. Survey findings should be interpreted in conjunction with these other efforts to provide an accurate, holistic, and nuanced understanding of the Massachusetts CHW workforce and trends.

Nonetheless, the results of the Massachusetts CHW Workforce Survey will provide critical insight into this diverse and important workforce as well as the impacts of certification. The survey design, sample frame development, survey administration, and statistical analysis is the culmination of over 5 years of effort, none of which would have been possible without our partners, CHW advocates, and CHW networks. Further, our conscientious design of the survey tools, rigorous documentation of sample frames, leveraging of local, state, and national CHW networks in outreach, and rigorous statistical analysis demonstrate how robust, replicable findings can be captured on this critical topic. Organizations interested in undertaking similar efforts can use, modify, and improve upon our tools and methods for their own assessments of the CHW workforce and their employers.

## Conclusion

There is a notable gap in the literature of studies rigorously evaluating state certification of CHWs. The results of our statistically weighted survey administered in a robustly defined sample frame before and after the launch of Massachusetts CHW certification will answer several important questions. These include whether state certification improves funding, job opportunities, and salaries for CHWs; whether there are potential adverse effects of state certification, such as inequitable access to certification, shifts in workforce demographics, employers adopting requirements for certification, or whether positive impacts are disproportionately benefiting certified CHWs; and whether state certification promotes the integration of CHWs into healthcare teams. Future research should assess similar measures leveraging longitudinal, observational study designs of individual CHWs to further elucidate the impacts of certification. Future research should also assess how CHW certification impacts health outcomes.

## Data availability statement

The datasets presented in this article are not readily available because these data are considered confidential by the Massachusetts Department of Public Health Privacy and Data Compliance Office (PDCO). Any data requests must follow processes and requirements of the Massachusetts Department of Public Health and PDCO. Requests to access the datasets should be directed to https://www.mass.gov/how-to/apply-for-access-to-mdph-confidential-records-data.

## Ethics statement

The studies involving human participants were reviewed and approved by University of Massachusetts Chan Medical School (UMass Chan) Institutional Review Board. Written informed consent from the patients/participants was not required to participate in this study in accordance with the national legislation and the institutional requirements.

## Author contributions

VN, GS, WWSU, and BB-C oversaw this research project. GH, TM, CS, EG, EM, CA, and BB-C provided subject matter expertise in survey design. VN, WWSU, P-PL, DB, and BB-C designed the statistical analysis plan. P-PL, DB, and BB-C collected and analyzed survey data. All authors contributed to the writing and editing of this manuscript. All authors contributed to the article and approved the submitted version.
