# Supplementary material for: Evaluating the impact of community health worker certification in Massachusetts: Design, methods, and anticipated results of the Massachusetts community health worker workforce survey
Source: Front Public Health. 2023 Jan 12;10:1043668. doi: 10.3389/fpubh.2022.1043668 (PMC9877511; doi:10.3389/fpubh.2022.1043668)
Supplement: Supplementary file 1 [file Table_1.PDF]

Supplement A. Evaluation Questions, Measures, and Survey Questions.

Evaluation Question	Measures with Hypothesized Direction	CHW Employer Survey	CHW Survey
1. Has certification increased opportunities for CHWs to have stable, better paid positions and best practice working conditions?	Increase in awareness of the CHW Board of Certification and increase in the proportion of certified versus non-certified CHWs.	(**EE7**) Before today, were you aware that there is a CHW Board of Certification in the state of Massachusetts?<br>(**EE8**) Are any CHWs employed by your organization certified by the MA CHW Board of Certification?<br>(**EE9**) Of the total number of CHWs employed by your organization, how many are certified by the MA CHW Board of Certification?	(**CD5**) Before today, were you aware that there is a CHW Board of Certification in the state of Massachusetts?<br>(**CD6**) Are you certified by the Massachusetts CHW Board of Certification?
	Increase in the number of CHWs that have jobs that are not paid by grants.	(**ED1**) How are CHWs at your organization funded?	
	Increases in CHWs salary pre- versus post-certification.	(**ED3**) What is the average annual salary of a CHW at your organization?<br>(**ED4**) Over the past 12 months, has the average annual salary of a CHW at your organization increased, decreased, or stayed the same?	(**CB6**) What is your income per year as a CHW at your organization?<br>(**CB7**) Over the past 12 months, has your income as a CHW at your organization increased, decreased, or stayed the same?
	Increase in proportion of employed CHWs that have jobs paid for out of organization's operating costs.	(**ED1**) How are CHWs at your organization funded?	
	Increase in proportion of employed CHWs that have jobs covered through payers.	(**ED2**) Are services provided by CHWs at your organization reimbursed by an insurer or other payer?<br>(**ED2a**) In which of the following ways are services provided by CHWs at your	

Evaluation Question	Measures with Hypothesized Direction	CHW Employer Survey	CHW Survey
		organization covered by an insurer or other payer?	
	Increase in the number of CHW jobs pre- and post-certification.	(**EB3**) How many of the following CHWs does your organization employ/are in the programs that you manage or work directly in?<br>(**EB4**) Does your organization currently have any job openings for CHWs?<br>(**EB5**) How many of each of the following job openings for CHWs does your organization have?	
	Increase in number of opportunities for CHW position promotions.	(**EC18e**) CHWs have opportunities for promotion at your organization.	(**CC15e**) I have opportunities for promotion at my organization.
2. Has certification changed the nature and qualities of the CHW workforce (e.g., race, ethnicity, education)?	Equal distribution of education attainment between certified CHWs and non-certified CHWs.		[*STRATA*] (**CD6**) Are you certified by the Massachusetts CHW Board of Certification?<br>(**CE3**) What is the highest grade or level of school that you have completed?
	Equal distribution of certified versus non-certified CHWs who are people of color, bilingual, or immigrants.		[*STRATA*] (**CD6**) Are you certified by the Massachusetts CHW Board of Certification?<br>(**CE1**) What is your age now?<br>(**CE2**) What is your gender?<br>(**CE4**) Are you of Hispanic or Latino origin or descent?<br>(**CE5**) What is your race?<br>(**CE6**) In which of the following languages are you fluent enough to

Evaluation Question	Measures with Hypothesized Direction	CHW Employer Survey	CHW Survey
			communicate with the individuals you serve as a CHW if you do not use an interpreter?
	Surveillance of certified and non-certified CHW employment in CBOs, health centers, and hospitals.	(**EA2**) Which of the following best describes the type of organization where you work? *(CBOs only)*<br><br>(**X***) Total CHW employers working in health center, CBO, and hospital settings.	(**CB2**) Which of the following best describes the type of organization you currently work for as a CHW? *(CBOs only)*<br>(**X***) Total CHWs working in health center, CBO, and hospital settings.
	Surveillance of CHW titles, activities, and services.	(**EC1**) Is CHWs' work at your organization related to any of the following specific health issues or chronic diseases?<br>(**EC2**) Please indicate how many of them are engaged in work related to each of the following health issues or chronic diseases.<br>(**EC3**) Which of the following health promotion and disease prevention related activities do CHWs at your organization engage in?<br>(**EC4**) How often do CHWs at your organization engage in each of the following activities?	(**CC1**) Is your work as a CHW at your organization related to any of the following specific health issues or chronic diseases?<br><br>(**CC2**) Which of the following health promotion and disease prevention related activities do you perform as a CHW at your organization?<br><br>(**CB3**) Which of the following is closest to your job title?
3. Has certification created different opportunities for certified and non-certified CHWs (e.g., type of employer,	Surveillance of the number of CHW jobs by employer certification requirements.	[*STRATA*] (**EE2**) Does your organization require CHWs to have any of the following qualifications to be hired?	

Evaluation Question	Measures with Hypothesized Direction	CHW Employer Survey	CHW Survey
salary, opportunities for training)?		(**EB3**) How many of the following CHWs does your organization employ/are in the programs that you manage or work directly in?<br>(**EB4**) Does your organization currently have any job openings for CHWs?<br>(**EB5**) How many of each of the following job openings for CHWs does your organization have?	
	Surveillance of employers that require certification versus those that do not.	(**EE2**) Does your organization require CHWs to have any of the following qualifications to be hired?	(**CD14**) Does your employer require CHW Board Certification?
	Surveillance of type of employer in which CHW and CHW employers work (i.e., CBO, hospital, health center) and by certification requirement and certification status.	[*STRATA*] (**EE2**) Does your organization require CHWs to have any of the following qualifications to be hired?<br>(**EA2**) Which of the following best describes the type of organization where you work? *(CBOs only)*<br><br>(**X***) Total CHW employers employed in health center, CBO, and hospital settings.	[*STRATA*] (**CD6**) Are you certified by the Massachusetts CHW Board of Certification?<br><br>(**CB2**) Which of the following best describes the type of organization you currently work for as a CHW? *(CBOs only)*<br>(**X***) Total CHWs employed in health center, CBO, and hospital settings.
	Surveillance of number of opportunities for CHW position promotions by certification requirement and certification status.	[*STRATA*] (**EE2**) Does your organization require CHWs to have any of the following qualifications to be hired?<br>(**EC18e**) CHWs have opportunities for	[*STRATA*] (**CD6**) Are you certified by the Massachusetts CHW Board of Certification?<br><br>(**CC15e**) I have opportunities for

Evaluation Question	Measures with Hypothesized Direction	CHW Employer Survey	CHW Survey
		promotion at your organization.	promotion at my organization.
	Equivalent increase in CHW salaries/ benefits (compensation package) among by certification requirement and certification status.	[*STRATA*] (**EE2**) Does your organization require CHWs to have any of the following qualifications to be hired?<br>(**ED3**) What is the average annual salary of a CHW at your organization?<br>(**ED4**) Over the past 12 months, has the average annual salary of a CHW at your organization increased, decreased, or stayed the same?<br>(**ED5**) Does your organization provide any of the following to CHWs?	[*STRATA*] (**CD6**) Are you certified by the Massachusetts CHW Board of Certification?<br><br>(**CB6**) What is your income per year as a CHW?<br><br>(**CB7**) Over the past 12 months, has your income as a CHW at your organization increased, decreased, or stayed the same?<br><br>(**CB8**) Do you receive any of the following from your organization?
	Equivalent access to CHW training among employers requiring CHW certification and employers not requiring CHW certification.	[*STRATA*] (**EE2**) Does your organization require CHWs to have any of the following qualifications to be hired?<br>(**EE6**) Does your organization provide any ongoing training or education to its CHWs?	
4. How accessible is the certification process for CHWs?	CHW perspectives on process of getting certified.		(**CD8**) How would you rate the process of getting certified?<br>(**CD9**) How likely are you to maintain or renew your CHW Board Certification?<br>(**CD10**) Select the top-3 reasons why you are [Somewhat unlikely/Very unlikely] to maintain or renew your CHW Board Certification?

Evaluation Question	Measures with Hypothesized Direction	CHW Employer Survey	CHW Survey
			(**CD11**) How interested are you in being a Board-Certified CHW?<br>(**CD12**) Select the top-3 reasons why you are [Somewhat uninterested/very uninterested] in being a Board-Certified CHW?
5. Has certification influenced how CHW employers value CHWs on their care team?	Increase in CHW employer and CHW perceived value of certification (health center and acute hospital only).	(**EE10**) Below are some statements regarding the value of CHW certification to your organization. Please indicate the extent to which you agree or disagree with each statement.	(**CD13**) Below are some statements regarding the value of CHW certification to you as a CHW. Please indicate the extent to which you agree or disagree with each statement.
	Increase in proportion of clinical employers that include CHWs in team meetings (health center and acute hospital only).	(**EC17a**) In which of the following ways do CHWs work directly with members of a clinical care team?	
	Increase in proportion of CHWs with access to and use of the electronic health record (health center and acute hospital only).	(**EC10**) Does your organization use patient electronic health records?<br>(**EC11**) Do CHWs at your organization have access to patients' electronic health records?<br>(**EC11a**) Which of the following are CHWs able to do with patients' electronic health records at your organization?<br>(**EC12**) Is CHWs' work at your organization included in patients' electronic health records?	(**CC8**) Does your organization use patient electronic health records?<br>(**CC9**) Do you have access to patients' electronic health records at your organization?<br>(**CC9a**) Which of the following are you able to do with patients' electronic health records at your organization?<br>(**CC10**) Are the services/activities you provide as a CHW included in patients' electronic health records?
	Increase in proportion of supervisors who	(**EC18b**) CHW's work is understood by the	(**CC15b**) My supervisor understands

Evaluation Question	Measures with Hypothesized Direction	CHW Employer Survey	CHW Survey
	understand CHW work (health center and acute hospital only).	individuals who supervise them.	the work I do as a CHW.
	Proportion of CHW employers that include CHWs in workflows pre-and post-certification (health center and acute hospital only).	(**EC17a**) In which of the following ways do CHWs work as part of a team at your organization?	

* CBO indicates community-based organization.

** Corresponding questions between the CHW and CHW employer surveys are aligned in the table.

*** "X" indicates that the data is not derived from a survey question.
