# Supplementary material for: Evaluating the impact of community health worker certification in Massachusetts: Design, methods, and anticipated results of the Massachusetts community health worker workforce survey
Source: Front Public Health. 2023 Jan 12;10:1043668. doi: 10.3389/fpubh.2022.1043668 (PMC9877511; doi:10.3389/fpubh.2022.1043668)
Supplement: Supplementary file 2 [file Data_Sheet_1.pdf]

Supplement B. CHW In-Depth-Interview Topic Guide
February 16, 2016

Note for Interviewer

This topic guide is for a qualitative conversation with CHWs to tap into their experiences and perceptions as a CHW, particularly with regard to their role within their organization, their work, training they have had or feel they need, and CHW certification. The purpose of this guide is to help gather baseline information to answer the following five evaluation questions:

1. Has Certification increased opportunities for CHWs to have stable, better-paid positions and working conditions?
2. Has Certification changed the nature and qualities of the CHW workforce (e.g., race, ethnicity, education)?
3. Has Certification created different opportunities for Certified and non-Certified CHWs (e.g., type of employer, salary, opportunities for training)?
4. How accessible is the Certification process for CHWs and approval process for training centers?
5. Has Certification influenced how CHW employers value CHWs on their care team?

DPH Definition (if needed)

DPH defines CHWs as public health workers who apply their unique understanding of the experience, language, and/or culture of the populations they serve in order to carry out one or more of the following roles:

- Providing culturally appropriate health education, information, and outreach in community-based settings, such as homes, schools, clinics, shelters, local businesses, and community centers;
- Bridging/culturally mediating between individuals, communities, and health and human services, including actively building individual and community capacity;
- Assuring that people access the services they need;
- Providing direct services, such as informal counseling, social support, care coordination, and health screenings; and
- Advocating for individual and community needs.

CHWs are distinguished from other health professionals because they:

- Are hired primarily for their understanding of the populations and communities they serve;
- Conduct outreach a significant portion of the time in one or more of the categories above;
- Have experience providing services in community settings.

Introduction (2-3 minutes)

- Introduce self
- State purpose – Thank you for taking the time to speak with me today. We are conducting these interviews on behalf of the Massachusetts Department of Public Health (DPH). DPH is interested in learning more about the experiences and perceptions of Community Health Workers and the work they do. DPH will use this information to improve training opportunities, and promote more stable and better jobs for Community Health Workers.

The interview with you and nine other CHWs will help us prepare a survey to be sent to CHWs around the state.

- I do want to mention that I would like to record this call so that I don't have to write down everything you say. The recording and any notes from the interview will not be shared with anyone outside the immediate research team, and nothing you say will be attributed in any report to you individually. Is it ok with you if I audiotape the interview?
- This call should take about 45 minutes. Do you have any questions before we begin?

Employment (5 minutes)

The purpose of this section is to help us understand the type of organization and program that you work in.

1. What is your job title? [if need explanation can provide details from box above and the following] CHW is an umbrella term, and CHWs have many different job titles. Some common ones are Patient Navigator, Community Health Advisor, Family Advocate, Outreach Worker, and many others.)

   Probe:

   a. What is it about your work now that makes you a community health worker?*

2. Please tell me a little bit about the organization where you work as a CHW.

   Probes:

   a. What type of organization do you work in?
   b. Do you work in a particular program within the organization?
   c. Is there more than one program that employs CHWs?
   d. About how many CHWs work at your organization?
      i. How many work in your program or area, if applicable?
   e. Do you work full time, part time… on a volunteer basis?
      i. If part time, how much? Weeks per year, hours per week, etc.
      ii. How long have you been in your current position?

3. How did you hear about your current position? e.g., referral, internet, etc.

   a. Did you have any trouble finding work as a CHW? Why/why not/explain

Work Description (15 minutes)

The next set of questions is to help us understand more about your day-to-day work.

4. I'd like to understand the work you do as a CHW. Please describe your typical responsibilities and walk me through your day.

   Probe

a. [If more than one activity] Do some activities take up more time than others? *(listen for information about the amount of documentation they do)*

b. In addition to the responsibilities and activities you described, do you help people manage their chronic disease? How? Which ones?

c. What other health conditions or patient needs do you work on regularly?

5. Do you work as part of a team(s) at your organization?

Probes

a. Who is on the team(s) with you?

b. How do you work with the team – *ask in context of primary care team or any other multi-disciplinary team the CHW works in.*

i. What is your role as a member of the team?

ii. Do you participate in team meetings? How often? Why/Why not?

iii. What level of access do you have, if any, to electronic health records (EHRs)? What kind of information do you enter into the EHR? If you don't enter information then who does? How do you keep track of your work?

6. Do you feel overall your organization values the work you do as a CHW? Why/why not?

Probes

a. Do you think your team members understand the work you do? Why/why not?

b. Do you think your team members value the work you do? Why/why not?

c. What aspect of your work do you think offers the greatest value to your team?*

d. Is your work supervised by someone? If yes, by whom?

i. Do you think your supervisor understands the work you do? Why/why not?

ii. Do you think your supervisor values the work you do? Why/why not?

iii. What type of supervision do you receive? How often? Does it help with your work? If not, what would be more helpful from your supervisor?

7. I would like to understand a little about how you are paid for your work as a CHW.

Probes

a. Are you a salaried employee? Or are you paid by the hour? (may need to explain salaried)

b. Has your income changed in the past 12 months? Increased/decreased/stayed the same?

8. Do you work for pay in another position besides your job as a CHW?

Probes

a. Why? E.g., volunteer as a CHW; work another job to supplement your income, etc.

9. **How long do you think you will continue to work as a CHW in the future?**

Probes

a. Why? Why not? Explain
b. What are your career goals? Do you want to continue working as a CHW?
c. What would help you meet your career goals as a CHW?

Training (5 minutes)

Next, I would like to discuss the type of training you have for your work as a CHW.

10. Tell me about any CHW-specific training you have.

    Probes

    a. Have you ever received any formal training as a community health worker?
    b. Where and when did you receive training? At a training center, on the job, both?
    c. Are you trained in certain topics? Core competencies? Special health topics? Both?

11. Has your employer helped you with your training? E.g., organized training sessions, reimbursed for training, allowed you to go to training during work time, etc.

    Probes

    a. Why (why not) do you think your employer has (or has not) helped?

12. Is there any particular type of training that you wish you had? What is the training?

    Probes

    a. What were the reasons for not getting this training? E.g., training not offered/available, poor access (not offered near you), time away from work, cost, etc.

Certification (10 minutes)

We are almost finished with the interview. The final section will ask questions about voluntary state certification for CHWs in Massachusetts.

13. Have you ever heard of voluntary state certification of CHWs in Massachusetts?
14. [If Yes] Please tell me what you know about it.
15. [If No, explain about the certification] As you may know, the legislature established a statewide CHW Board of Certification at the Department of Public Health in 2012. Certification for CHWs is voluntary but the idea behind certification is to better define the work that CHWs perform, to establish training standards, to strengthen awareness and understanding and value of CHWs in public health and health care, and to ultimately promote job creation and stable funding for CHWs.
16. **How do you think certification could affect the CHW profession?**

    Probes

    a. Do you see any potential plusses and minuses for you as a CHW?
    b. How could it affect employers?

    c. How could it potentially affect CHWs?

17. Renewal of certification will likely require 15 hours of continuing education every two years. What do you think of this requirement? Explain.

Background/Demographics (5 minutes)

18. And to wrap up, it would be great if you can give me a little background information about yourself.

    Probes

    a. For how many years have you been working as a CHW in the U.S.?
    b. How old are you? Are you 18-24 / 25-44 / 45-64 / 65+
    c. What is the highest grade or level of school you have completed? (8th grade or less / Some high school / High school graduate or GED / Some college / 4-year college degree/ More than 4 year college degree)
    d. Are you of Hispanic or Latino origin or descent?
    e. What is your race? (White / Black or African American / Asian / Native Hawaiian or other Pacific Islander / American Indian or Alaska Native / Other)
    f. And which ethnicity do you most identify with?

19. Other than English, would you say you are fluent in any other language to communicate with the individuals you serve as a CHW?

Wrap-Up (2 minutes)

20. Anything else you would like to tell me that we haven't touched upon that you think might be relevant for DPH to hear about your experience as a CHW?
21. Thank you so much for your time and the information you have provided. I'd like to confirm your email address so we can send you a gift card as a token of our appreciation.
