# Supplementary material for: Evaluating the impact of community health worker certification in Massachusetts: Design, methods, and anticipated results of the Massachusetts community health worker workforce survey
Source: Front Public Health. 2023 Jan 12;10:1043668. doi: 10.3389/fpubh.2022.1043668 (PMC9877511; doi:10.3389/fpubh.2022.1043668)
Supplement: Supplementary file 3 [file Data_Sheet_2.pdf]

Supplement C. CHW Employer In-Depth-Interview Topic Guide
February 16, 2016

Note for Interviewer

This topic guide is for a qualitative conversation with employers to discuss their experiences and perceptions of CHWs, particularly with regard to their roles and duties within their organization, their overall work, training, and CHW certification. The purpose of this guide is to help gather baseline information to answer the following four evaluation questions:

1. Has Certification increased opportunities for CHWs to have stable, better-paid positions and working conditions?
2. Has Certification changed the nature and qualities of the CHW workforce (e.g., race, ethnicity, education)?
3. Has Certification created different opportunities for Certified and non-Certified CHWs (e.g., type of employer, salary, opportunities for training)?
4. Has Certification influenced how CHW employers value CHWs on their care team?

DPH Definition (if needed)

DPH defines CHWs as public health workers who apply their unique understanding of the experience, language, and/or culture of the populations they serve in order to carry out one or more of the following roles:

- Providing culturally appropriate health education, information, and outreach in community-based settings, such as homes, schools, clinics, shelters, local businesses, and community centers;
- Bridging/culturally mediating between individuals, communities, and health and human services, including actively building individual and community capacity;
- Assuring that people access the services they need;
- Providing direct services, such as informal counseling, social support, care coordination, and health screenings; and
- Advocating for individual and community needs.

CHWs are distinguished from other health professionals because they:

- Are hired primarily for their understanding of the populations and communities they serve;
- Conduct outreach a significant portion of the time in one or more of the categories above;
- Have experience providing services in community settings.

Introduction (2 minutes)

- Introduce self
- State purpose – Thank you for taking the time to speak with me today. We are conducting these interviews on behalf of the Massachusetts Department of Public Health (DPH). DPH is interested in learning more about employers' experiences and perceptions of Community Health Workers and the work they do. DPH will use this information for improving workforce development and increased integration of Community Health Workers into care teams. The interview with you and nine other CHW organizations will help us prepare a survey to be sent to employers of CHWs around the state.

- I do want to mention that I would like to record this call so that I don't have to write down everything you say. The recording and any notes from the interview will not be shared with anyone outside the immediate research team, and nothing you say will be attributed in any report to you individually. Is it ok with you if I audiotape the interview?
- This call should take about 45 minutes. Do you have any questions before we begin?

Background (5 minutes)

The purpose of this first section is to help us understand the type of organization and program that you are working in.

1. Please tell me a little bit about your organization.

   Probes:

   a. What type of organization do you work at? *[If multi-site, ground the respondent to always answer for the site they're at.]*
   b. About how many employees, including yourself, does your organization currently employ? Full time/part time
   c. Can you provide an estimate of how many clients (e.g., patients or individuals) does your organization serve each year?
   d. What is your job title?
   e. What are your primary roles in the organization? Do you work directly with CHWs? What is the nature of your working relationship with CHWs?*

Current CHW Roles/Job Duties (15 minutes)

Thank you, the next section of the interview will focus on the type of work CHWs in your organization do.

2. What job title(s) do CHWs have at your organization? ([if needed] CHW is an umbrella term, and CHWs have many different job titles. Some common ones are Patient Navigator, Community Health Advisor, Family Advocate, Outreach Worker, and many others.)
3. About many CHWs currently work at your organization (Or site? Or program?)? Full time, part time, volunteer basis.

   Probes:

   a. Are they concentrated in particular programs, departments, or divisions?
4. Can you tell me about the typical roles and functions CHWs perform at your organization/site/program?

   Probes:

   a. [If more than one activity] what are the most common kinds of roles CHWs play and services they provide?
      i. Are CHWs in your organization engaged in chronic disease prevention or management activities? Which activities?

ii. Do CHWs in your organization link or refer clients (e.g., patients or consumers) to outside resources or services? If so what types of resources/resource guides or tools?

b. Do your CHW programs focus on priority populations? If so what populations (e.g., geographic area, gender, race/ethnicity, disease specific programming)?

c. What is a typical caseload for a CHW at your organization (i.e., what is the average number of clients they have at a given time)? Do you think the caseload is manageable? Explain.

5. Do CHWs work as part of a team(s) at your organization?

   Probes:

   a. Who is on the team with CHWs?

      i. [If part of a primary care team] What is the makeup of care teams/who are members of your care teams/which clinical and non- clinical providers are part of care teams?

   b. Tell me about the ways CHWs work within the team – *ask in context of primary care team or any other multi-disciplinary team CHWs work in.*

      i. What is the CHW's role on the team?

      ii. Do CHWs participate in team meetings? How often? Why/why not?

      iii. Do you think CHWs' work is understood by the teams they work with? Why/why not?

      iv. Do you think CHWs' work is valued by the teams they work with? Why/why not?

      v. What level of access do CHWs have, if any, to patients' electronic health records (EHRs)? What information related to CHWs' work is included in patient's EHRs?

      vi. Please describe any lessons learned from integrating CHWs into the team(s).

6. Who directly supervises the CHWs within your program/organization? (position/professional background)

   Probes:

   a. Do you think the individuals who supervise CHWs understand their work? Why/why not? (if they are the supervisor then ask the question to them directly and probe to focus on how they came to understand their work and promote it within the organization)

   b. Do you think the individuals who supervise CHWs value their work? Why/why not? (if they are the supervisor then ask the question to them directly and probe to focus on how they came to value their work and promote it within the organization)

CHW Advantages and Barriers (7 minutes)

The next few questions will focus on why CHWs are part of your organization.

7. **What do you think is the greatest value that CHWs provide to your organization?**

   Probes:

   a. Why do you employ them?
   b. Why is that valuable to you? What do CHWs add that other team members don't?
   c. How effective are they at what they do? Please explain.

8. **Can you please describe how CHWs are funded in your organization?**

   Probe

   a. 100% grant funded, partially grant funded, global third-party payment, etc.
   b. What are sources of the grants (foundation, federal, state, local)?
   c. How long do you expect the funding for CHWs to last at in your organization/program?

9. Can you tell me a bit about how CHWs are paid in your organization?

   Probes

   a. What is the average salary of a CHW at your organization?
      i. How have levels been determined and how would they be determined in the future?
      ii. Has the average salary changed in the past 12 months?
   b. What employee benefits, if any, are CHWs in your organization eligible to receive? E.g., health insurance, vacation accrual, paid leave for training, tuition remission etc.

10. What are your organization's future plans for CHWs?

    Probes

    a. Do you think your program/organization will continue to support CHWs? How?
    b. Do you think your program/organization will hire additional CHWs?
       i. Why?
       ii. Why not?
       iii. [If funding is a deterrent] Assuming your organization had funding available for CHWs, would you be interested in growing your CHW workforce? Why/ Why not?
    c. What do you think would help to recruit and retain CHWs at your organization? Are you currently doing anything to recruit and retain CHWs?

CHW Training (7 minutes)

The next section is focused on the training CHWs receive in your organization and outside of your organization.

11. When you are recruiting CHWs, what qualities do you look for?

    Probes

    a. Is there any specific training, education, qualifications, other experiences?

12. Tell me a little bit about the training that CHWs that work at your organization have.

    Probes

    a. Do they receive any CHW-specific training (such as core competency training from training centers outside of your organization)? If yes…
        i. From where and when have they received training? (within organization or outside of organization)
        ii. Which topic areas are CHWs trained in?
    b. Does your organization provide any in-house training or education to the CHWs you employ? Why/why not.
        i. [If yes] How frequently do you provide training or education?

13. What opportunities for advancement are available to CHWs at your organization?
    a. Are there different tiers/levels of CHW positions?
    b. Do you recognize CHWs for their experience?
    c. How often are CHWs promoted?
    d. What types of positions are available?

Certification (7 minutes)

We are almost finished with the interview. The final section will ask questions about voluntary state certification for CHWs in Massachusetts.

14. Have you ever heard of voluntary state certification of CHWs in Massachusetts?
15. [If Yes] Please tell me what you know about it.
16. [If No, explain about the certification] As you may know, the legislature established a statewide CHW Board of Certification at the Department of Public Health in 2012. Certification for CHWs is voluntary but the idea behind certification is to better define the work that CHWs perform, to establish training standards, to strengthen awareness and understanding and value of CHWs in public health and health care, and to ultimately promote job creation and stable funding for CHWs.
17. **How do you think certification will impact employers' perceptions of CHWs?**

    Probes

    a. Would certification make you more likely to hire CHWs? Why or why not?

b. Can you see any downsides to certification? What are they?
c. How do you think certification could affect the CHW profession, both positively or negatively?

Wrap-Up (2 minutes)

18. Anything else you would like to tell me that we haven't touched upon that you think might be relevant for DPH to hear about your experience as a CHW employer?
19. Thank you so much for your time and the information you have provided. I'd like to confirm your email address so we can send you a gift card as a token of our appreciation.
