# Supplementary material for: Evaluating the impact of community health worker certification in Massachusetts: Design, methods, and anticipated results of the Massachusetts community health worker workforce survey
Source: Front Public Health. 2023 Jan 12;10:1043668. doi: 10.3389/fpubh.2022.1043668 (PMC9877511; doi:10.3389/fpubh.2022.1043668)
Supplement: Supplementary file 4 [file Data_Sheet_3.pdf]

Supplement D. CHW Employer Survey
W2 Final Version: 3/18/21

Notes on how to read this survey document:

- Text in **BOLD CAPS** in brackets represents programming instructions, which will not be visible in the online survey.
- **SP** means "single punch" where only one response is allowed from the list of response options and **MP** means "multi punch" where more than one response can be selected.
- Question numbers or response list numbers will not visible in the online survey.

[SHOW ALL] [INTRO A]

Massachusetts Department of Public Health
2021 Community Health Worker Employer Survey

Thank you for participating in the Community Health Worker (CHW) Employer Survey being conducted by the Massachusetts Department of Public Health (DPH). DPH is conducting this survey to learn more about how employers view and work with Community Health Workers (CHWs) in their organization so that we can better support CHW workforce development. Your participation is greatly appreciated.

Please note the following:

- The words "your organization" in this survey refer to **[INSERT SITE NAME]**.
- The acronym "CHWs" is used in this survey to refer to Community Health Workers.

[ASK ALL] [SP] [REQUIRE ANSWER] [PROMPT IF LEFT BLANK: You must respond to this question in order to proceed with the survey.**]**

EA1. Our records indicate that you oversee or work directly with Community Health Workers (CHWs). CHWs are known by many titles, including Outreach Worker, Patient Navigator, Community Health Advisor, Peer Navigator, Community Health Representative, Community Health Advocate, Promotora, Family Advocate, and many other names.

Please confirm that you are a CHW program manager/director or someone who employs, oversees or works directly with CHWs at your organization.

1 Yes
2 No

[TERMINATE IF EA1=2 (NO)]
Thank you for your time. You are not eligible to take this survey.

[ASK IF ORGANIZATION_TYPE = CBO AND EA1=1 (Yes)] [MP]

EA2. Which of the following describe the type of organization where you work? *Please select all that apply.*

1 Community Action Agency
2 Disability Services
3 Domestic Violence Services
4 Elder Services
5 HIV/AIDS Services
6 Housing Support
7 Rape Crisis
8 Legal Services
9 Refugee/Immigrant
10 Substance Use Services
11 Other Mental/Behavioral Health Services
12 Teen Health
13 Veteran Services
14 Other (Please specify ________________________)

[ASK IF EA1=1 (YES)] [SP]

EA3. Which of the following **is closest to** your job title? *Please select one response.*

1 Clinical/Medical Director
2 Executive Director
3 Practice Manager
4 Program Manager/Director
5 Project Coordinator
6 Project Director
7 Team Leader
8 Volunteer Coordinator
9 Community Health Worker (CHW)
10 CHW Supervisor
11 Senior CHW
12 Case Manager
13 Nurse Manager
14 Social Worker
15 Behavioral health care provider (e.g., psychiatrist, mental health counselor, etc.)
16 Other (Please specify__________________)

[SHOW IF EA1=1 (YES)] [INTRO B]

Community Health Workers at your Organization

The next few questions are about Community Health Workers (CHWs) at your organization. As a reminder, by "your organization" we are referring to **[INSERT SITE NAME]**.

[ASK IF EA1=1 (YES)] [SP] [PROMPT IF LEFT BLANK: We would really like to have your answer to this question.**]**

EB1. Do any Community Health Workers (CHWs) currently work for your organization?

1 Yes

2 No

[ASK IF EB1=1 (YES)] [NUMERIC BOX] [RANGE = 1-20. IF "0" PROMPT: You indicated that you are a CHW program manager/director or someone who employs, oversees or works directly with CHWs at your organization. Please adjust your answer.**] [IF RESPONSE EXCEEDS RANGE, PROMPT:** You have entered a very large number. Please double-check your response.**]**
[PROMPT IF LEFT BLANK: We would really like to have your answer to this question.**]**

EB2. Please think of the departments or programs at your organization **that employ CHWs**. How many such departments or programs do you manage or work directly in?

___________ departments or programs

[ASK IF EB1=1 (YES)] [NUMERIC BOXES] [RANGE FOR TOTAL = 1-19. IF "0" PROMPT: You indicated that CHWs currently work for your organization. Please adjust your answer. **IF TOTAL EXCEEDS RANGE, PROMPT:** You have entered a very large number of CHWs; please double-check your response.**]**
[PROMPT IF LEFT BLANK: We would really like to have your answer to this question. Please provide your best estimate.**]**

EB3. **[IF EB2=1]** How many of the following CHWs does your organization employ? **[IF EB2>1 OR BLANK OR 0]** How many of the following CHWs are in the programs that you manage or work directly in?

Full time (at least 30 hours per week), paid CHWs	_________
Part time (less than 30 hours per week), paid CHWs	_________
Volunteer CHWs, unpaid	_________
**Total CHWs**	_________ **[SHOW RUNNING TOTAL]**

[SHOW IF EB2 > 1] [INSTRUCTION]
For all the remaining questions in this survey, the term "your organization" refers to ***only those programs that you manage or work directly in.***

[ASK IF EB1=1 (YES)] [SP]

EB4. Does your organization currently have any job openings for CHWs?

1 Yes

2 No

[ASK IF EB4=1 (YES)] [NUMERIC BOXES; RANGE FOR TOTAL = 1-19. IF "0" PROMPT: You indicated that your organization currently has job openings for CHWs. Please adjust your answer. **IF**

RESPONSE EXCEEDS RANGE, PROMPT: You have entered a very large number of job openings; please double-check your response.]

EB5. How many of each of the following job openings for CHWs does your organization have?

Full time (at least 30 hours per week), paid CHWs	_________
Part time (less than 30 hours per week), paid CHWs	_________
Volunteer CHWs, unpaid	_________
**Total job openings**	_________ **[SHOW RUNNING TOTAL]**

[SHOW IF EB1=1 (YES)] [INTRO C]

The Role of CHWs at your Organization

The next few questions are about the responsibilities and activities of Community Health Workers (CHWs) at your organization.

[ASK IF EB1=1 (YES)] [MP] [PROMPT IF LEFT BLANK: We would really like to have your response to this question.]

EC1. Is CHWs' work at your organization related to any of the following **specific health issues or chronic diseases**? *Please select all that apply.*

1 Asthma
2 Cancer
3 Cholesterol management
4 COVID-19
5 Dementia/Alzheimer's
6 Diabetes
7 Disabilities (e.g., physical, developmental)
8 Falls prevention
9 Heart disease
10 Hepatitis (Hep B, Hep C)
11 HIV/AIDS
12 Hypertension
13 Maternal and infant health
14 Obesity
15 Oral health
16 Sexual health
17 Substance use
18 Other Mental/Behavioral health
19 Tobacco cessation
20 Tuberculosis
21 Other health issue/chronic disease (Please specify ____________)

22 *None of the above* [SP]

[ASK IF EB3 TOTAL > 1 AND EC1 = ANY OF 1-21)] [SHOW ITEMS SELECTED IN EC1] [NUMERIC RESPONSES, RANGE FOR EACH ITEM = 1 TO VALUE IN EB3 TOTAL] [PROMPT IF ANY ITEM IS BLANK, ZERO OR EXCEEDS RANGE: 'One or more of your responses is outside of the valid range (1 to "EB3total"). Please double-check your answer.'**]**

EC2. You mentioned that you employ **[INSERT VALUE FROM EB3 TOTAL**] CHWs at your organization. Please indicate how many of them are engaged in work related to each of the following health issues or chronic diseases.

[INSERT ITEMS SELECTED IN EC1 WITH NUMBERIC BOXES FOR EACH ITEM]

[ASK IF EB1=1 (YES)] [] [MP] [PROMPT IF LEFT BLANK: We would really like to have your answer to this question**.]**

EC3. Which of the following **health promotion and disease prevention related activities** do CHWs at your organization engage in? *Please select all that apply.*

1 Care coordination (e.g., referrals to medical or community-based services)
2 Case management
3 Chronic disease self-management (e.g., Diabetes Prevention Program, My Life My Health, etc.)
4 Communicating between providers and patients/families
5 Contact tracing for COVID-19
6 Other COVID-19 related activities
7 Teaching health literacy
8 Individual or community needs assessment
9 Individual or community outreach and education
10 Informal support/counseling
11 Medical appointments (e.g., scheduling, maintaining, etc.)
12 Helping patients with medication/treatment adherence
13 Motivational interviewing
14 Promoting healthy lifestyles (e.g., nutrition, exercise, etc.)
15 Support groups (e.g., organizing, leading)
16 Assisting with transitions of care (e.g., post hospital discharge)
17 Other health promotion activity (Please specify _______________)
18 *None of the above* [SP]

[ASK IF EC3 = ANY OF 1-17)] [SHOW ITEMS SELECTED IN EC3 USING SAME ORDER] [GRID: SP ACROSS, MP DOWN]

EC4. How often do CHWs at your organization engage in each of the following activities?

	Very often 4	Often 3	Sometimes 2	Rarely 1
[INSERT ITEMS SELECTED IN EC3]				

[ASK IF EC3=3 (CHRONIC DISEASE SELF-MANAGEMENT)] [MP]

EC5. Do CHWs at your organization link or refer individuals to/from any of the following **chronic disease management community resources**? *Please select all that apply.*

1 Diabetes Prevention Program (DPP)
2 Diabetes Self-Management Education Program (DSME)
3 Chronic Disease Self-Management Education Program (CDSME)/My Life My Health
4 Blood Pressure Self-Management Program
5 Other chronic disease management community resource (Please specify____________)
6 *None of the above* **[SP]**

[ASK IF EB1=1 (YES)] [MP]

EC6. Which of the following **services** do CHWs at your organization connect clients or patients to? *Please select all that apply.*

1 Child care
2 Clothing and other household goods
3 Education assistance/resources
4 Employment
5 Food security
6 Fuel/utility assistance
7 Health insurance enrollment
8 Housing
9 Income assistance
10 Legal services
11 Long-term services and supports (e.g., home care, adult day programs)
12 Translation/interpretation
13 Transportation
14 Violence prevention (e.g., shelter)
15 Payment assistance for medication
16 Support with mental/behavioral health
17 Other non-medical service (Please specify ____________________)

18 *None of the above* [SP]

[ASK IF EB1=1 (YES)] [GRID: SP ACROSS, MP DOWN]

EC6a. Thinking of all the work CHWs employed by your organization perform, how much of their time do they spend at each of the following locations? *Please select a response for each item.*

	None of their time 1	Some of their time 2	Most of their time 3
a. Your organization's location			
b. At another organization's location			
c. In the community			

[ASK IF EB1=1 (YES)] [MP]

EC7. Which of the following **special populations** do CHWs at your organization work with? *Please select all that apply.*

1 Children/adolescents
2 Farm workers
3 Foreign nationals/immigrants/refugees
4 History of frequent Emergency Department (ED) use
5 History of frequent hospitalization
6 Homeless individuals
7 Individuals without a Primary Care Provider
8 Isolated rural residents
9 Seniors (ages 65 and up)
10 Sexual minorities (i.e., LGBTQ individuals)
11 Pregnant women and infants
12 Uninsured individuals
13 Other special populations (Please specify ____________________)
14 *None of the above* [SP]

[ASK IF EB1=1 (YES)] [SP]

EC8. Do CHWs at your organization mostly work with **specific race/ethnicity groups**?

1 Yes
2 No

[ASK IF EC8=1 (YES)] [MP]

EC9. Which **specific race/ethnicity groups** do CHWs at your organization mostly work with? *Please select all that apply.*

1 Black or African American
2 Hispanic or Latino
3 Asian
4 American Indian or Alaska Native
5 Native Hawaiian or Other Pacific Islander
6 White
7 Other race/ethnic group (Please specify ________________)

[ASK IF EB1=1 (YES)] [SP]
EC10. Does your organization use patient electronic health records (EHRs)?

1 Yes
2 No

[ASK IF EC10=1 (YES)] [SP]
EC11. Do CHWs at your organization have access to patients' electronic health records (EHRs)?

1 Yes
2 No

[ASK IF EC11=1 (YES)] [MP]
EC11a. Which of the following are CHWs able to do with patients' electronic health records (EHRs) at your organization? *Please select all that apply.*

1 Read the patient registry list
2 Enter notes into a patient record
3 Look up information on the patient record (e.g., needed/completed tests, appointments kept/missed, etc.)
4 Schedule appointments
5 Other (Please specify ________________)

[ASK IF EC10=1 (YES)] [SP]
EC12. Is CHWs' work at your organization included in patients' electronic health records (EHRs)?

1 Yes
2 No

[ASK IF EB1=1 (YES)] [SP]
EC13. Do CHWs at your organization receive supervision?

1 Yes
2 No

[ASK IF EC13=1 (YES)] [SP]
EC14. Do you supervise any of the CHWs at your organization?

1 Yes
2 No

[ASK IF EC14=2 (NO)] [MP]
EC15. Who supervises the CHWs at your organization? *If there is more than one supervisor, please select all that apply.*

1 Clinical/Medical Director
2 Executive Director
3 Practice Manager
4 Program Manager/Director
5 Project Coordinator
6 Project Director
7 Team Leader
8 Volunteer Coordinator
9 Community Health Worker (CHW)
10 Senior CHW
11 Case Manager
12 Nurse Manager
13 Social Worker
14 Behavioral health care provider (e.g., psychiatrist, mental health counselor, etc.)
15 Other (Please specify_________________)

[ASK IF ORGANIZATION_TYPE = HOSPITAL OR CHC AND EB1=1 (YES)] [SP] [PROMPT: We would really like to have your response to this question.**]**
EC16a.Do CHWs at your organization **work directly with members of a clinical care team** (i.e., nurses, social workers, primary care providers, mental/behavioral health providers)?

1 Yes
2 No

[ASK IF EC16a=1 (YES)] [MP] [PROMPT: We would really like to have your response to this question.**]**
EC16b. Which of the following types of members of a clinical care team do CHWs **work directly with** at your organization? *Please select all that apply.*

1 Behavioral health care provider (e.g., psychiatrist, mental health counselor, etc.)
2 Primary care provider (physician, nurse practitioner, physician's assistant)

3 Specialist (e.g., oncologist, cardiologist, OB-GYN, etc.)
4 Registered nurse
5 Social worker
6 Other clinical providers (Please specify _______________)

[ASK IF EC16a=1 (YES)] [MP]
EC17a. In which of the following ways do CHWs **work directly with members of a clinical care team**? *Please select all that apply.*

1 Participate in regular meetings
2 Meet on an as-needed basis
3 Receive patient or client referrals
4 Help develop care plans
5 Help implement care plans
6 Participate in case reviews
7 **[SHOW IF EC11=1 (YES)]** Document patient encounters in the same electronic health record (EHR) as other members of the clinical care team
8 Other (Please specify _______________)

[ASK IF EB1=1 (YES)] [SP]
EC17b. Do CHWs at your organization communicate about patients or clients regularly with administrative staff?

1 Yes
2 No

[ASK IF EB1=1 (YES] [GRID: SP ACROSS, MP DOWN]
EC18. Thinking of the CHWs at your organization, please indicate how much you agree or disagree with each of the following statements.

	Agree completely 4	Agree somewhat 3	Disagree somewhat 2	Disagree completely 1
a. Your organization values the work that CHWs do				
b. **[IF EC13=1 (YES)]** CHWs' work is understood by the individuals who supervise them				
c. CHWs' work is understood by the teams they work with				
d. CHWs are valued members of the teams they work with				
e. CHWs have opportunities for promotion at your organization				

[SHOW IF EB1=1 (YES)] [INTRO D]

CHW Compensation

The next few questions are about the compensation that Community Health Workers (CHWs) at your organization receive.

[ASK IF EB1=1 (YES)] [MP]
ED1. How are CHWs at your organization funded? *Please select all that apply.*

1 Your organization's core operating funds
2 Grant funding – Federal
3 Grant funding – State
4 Grant funding – Private
5 ACO financing/funding
6 Managed care financing
7 Other (Please specify ________________)
8 Not sure **[SP]**

[ASK IF EB1=1 (YES)] [SP]
ED2. Are services provided by CHWs at your organization covered by an insurer or other payer?

1 Yes
2 No

[ASK IF ED2=1 (YES)] [MP]
ED2a. In which of the following ways are services provided by CHWs at your organization covered by an insurer or other payer?

1 Through direct billing of CHW services
2 Through global payments
3 Through grant funding
4 Other way (Please specify ________________)

[ASK IF EB1=1 (YES)] [SP]
ED3. What is the **average annual salary** of a CHW at your organization? *If CHWs are paid an hourly rate, please provide your best estimate of their annual salary.*

1 Less than $15,000 a year
2 $15,000 to less than $20,000 a year
3 $20,000 to less than $30,000 a year
4 $30,000 to less than $40,000 a year
5 $40,000 to less than $50,000 a year
6 $50,000 to less than $60,000 a year
7 $60,000 to less than $75,000 a year

8 $75,000 a year or higher

[ASK IF EB1=1 (YES)] [SP]

ED4. Over the past 12 months, has the average annual salary of a CHW at your organization increased, decreased, or stayed the same?

1 Increased
2 Decreased
3 Stayed the same

[ASK IF EB1=1 (YES)] [MP]

ED5. Does your organization provide any of the following to CHWs? *Please select all that apply. If none of these are provided, please select "none of the above."*

1 Child care
2 Commuter subsidy
3 Unpaid leave for training/education
4 Paid leave for training/education
5 Health/dental insurance
6 Mileage/parking reimbursement
7 Paid time off (vacation, sick time, personal time, etc.)
8 Pension or retirement plan
9 Tuition assistance
10 Wage/salary increase
11 Other benefit (Please specify ________________)
12 *None of the above* **[SP]**

[ASK IF EA1=1 (YES)] [MP]

ED6. Which of the following might be reasons that are preventing your organization from hiring **[IF EB1=1, PIPE-IN:** additional] CHWs? *Please select all that apply.*

1 Lack of funding
2 Inability to bill insurers for CHW services
3 Lack of clarity on their scope of work
4 Lack of clarity about their value
5 Lack of clarity on which qualifications to look for
6 Lack of clarity on how to integrate them with other teams
7 Lack of training for CHWs
8 Lack of certification of CHWs
9 Lack of qualified applicants

10 Other (Please specify ___________________)
11 *None of the above – do not need* [IF EB1=1, PIPE-IN: *additional*] *CHWs* [SP]

[SHOW IF EB1=1 (YES)] [INTRO E]

CHW Training

The next few questions are about the education or training that Community Health Workers (CHWs) at your organization have or receive.

[ASK IF EB1=1 (YES)] [SP]
EE1. What minimum educational requirement, if any, must CHWs meet to be hired at your organization? *Please select one response.*

1 No minimum educational requirement
2 High school diploma/GED
3 Some college
4 Associate's degree
5 Bachelor's degree
6 Master's degree

[ASK IF EB1=1 (YES)] [MP]
EE2. Does your organization require CHWs to have any of the following qualifications to be hired? *Please select all that apply.*

1 Licensed health care professional (RN, LICSW or other licensed health professional)
2 State Certified by the Massachusetts CHW Board of Certification
3 Completed 80 hours of CHW core competency training
4 None of the above [SP]
5 Don't know [SP]

[ASK IF EB1=1 (YES)] [MP]
EE3. Which of the following does your organization look for when hiring a CHW? *Please select all that apply.*

1 Bilingual or multi-lingual
2 Prior experience as a CHW
3 Prior experience with the population served
4 Shared background with the population served
5 Social services background
6 Healthcare background
7 Knowledge of community services or resources
8 Valid driver's license for work-related travel
9 Own car for work-related travel
10 Other (Please specify_______________)
11 *None of the above* [SP]

[ASK IF EB1=1 (YES)] [MP]

EE4. Do CHWs at your organization have training related to any of the following **specific health issues or chronic diseases**? *Please select all that apply.*

1 Asthma
2 Cancer
3 Cholesterol management
4 COVID-19
5 Dementia/Alzheimer's
6 Diabetes
7 Disabilities (e.g., physical, developmental)
8 Falls prevention
9 Heart disease
10 Hepatitis (Hep B, Hep C)
11 HIV/AIDS
12 Hypertension
13 Maternal and infant health
14 Obesity
15 Oral health
16 Sexual health
17 Substance use
18 Other Mental/Behavioral health
19 Tobacco cessation
20 Tuberculosis
21 Other health issue/chronic disease (Please specify ____________)
22 *None of the above* **[SP]**

[ASK IF EB1=1 (YES)] [MP]

EE5. Which of the following types of training or education for CHWs does your organization support? *Please select all that apply.*

1 80 hours of CHW core competency training
2 Chronic disease self-management (e.g., Diabetes Prevention Program, My Life My Health, etc.)
3 Motivational interviewing
4 Medical interpreting training
5 Patient navigation/Navigator training
6 Emergency response training (e.g., first aid, CPR, etc.)
7 Other CHW-specific training (Please specify ______________)
8 *None of the above* **[SP]**

[ASK IF EB1=1 (YES)] [SP]

EE6. Does your organization provide any ongoing training or education to its CHWs?

1 Yes
2 No

[ASK IF EB1=1 (YES)] [GRID: SP ACROSS, MP DOWN]

EE6a. Has your organization provided health and safety training to CHWs on the following topics?

	Yes<br>1	No<br>2	Not sure<br>3
a. Hazards on the job			
b. Workers' rights under Occupational Safety and Health Administration (OSHA)			
c. Workers' Compensation			

[ASK IF EA1=1 (YES)] [SP] [PROMPT IF LEFT BLANK: We would really like to have your response to this question.**]**

EE7. Before today, were you aware that there is a CHW Board of Certification in the state of Massachusetts?

1 Yes
2 No
3 Not sure

[ASK IF EB1=1 (YES) AND EE7=1 (YES)] [SP]

EE8. Are any CHWs employed by your organization certified by the Massachusetts CHW Board of Certification?

1 Yes
2 No
3 Not sure

[ASK IF EB3 TOTAL>1 AND EE8=1 (YES)] [NUMERIC BOX, RANGE = 1 TO VALUE IN EB3 TOTAL] [IF RESPONSE EXCEEDS RANGE, PROMPT: Your response is outside of the valid range. Please double-check your answer.] **[PROMPT IF LEFT BLANK:** We would really like to have your response to this question. Please provide your best estimate.**]**

EE9. Of the **[INSERT VALUE FROM EB3 TOTAL]** CHWs employed by your organization, how many are certified by the Massachusetts CHW Board of Certification?

____

[ASK IF EA1=1 (YES)] [GRID: SP ACROSS, MP DOWN]

EE10. *As you may know, a Massachusetts CHW Board of Certification was established at the Department of Public Health in 2012 to develop a process for voluntary certification of CHWs. The purpose is to better define CHWs' work, establish training standards, and promote job creation and stable funding for CHWs. Certification became available in October 2018 (experience pathway only).*

Below are some statements regarding the **value of CHW certification to your organization**. Please indicate the extent to which you agree or disagree with each statement.

CHW certification will <u>help your organization</u> by…	Agree completely 4	Agree somewhat 3	Disagree somewhat 2	Disagree completely 1
a. Better defining the role of CHWs				
b. Helping CHWs learn new skills				
c. Improving CHWs' work performance				
d. Expanding CHW responsibilities				
e. Winning CHWs more respect from the individuals they serve				
f. Winning CHWs respect from other professionals				
g. Better integrating CHWs with other teams				
h. Helping obtain more stable funding for CHWs				
i. Enabling better coverage of CHW work through insurance or other payer				
j. Increasing CHWs' opportunities for promotion within your organization				

[THANK AND CLOSE]
Thank you. Please click the "OK" button to submit your responses.
