# Supplementary material for: Evaluating the impact of community health worker certification in Massachusetts: Design, methods, and anticipated results of the Massachusetts community health worker workforce survey
Source: Front Public Health. 2023 Jan 12;10:1043668. doi: 10.3389/fpubh.2022.1043668 (PMC9877511; doi:10.3389/fpubh.2022.1043668)
Supplement: Supplementary file 5 [file Data_Sheet_4.pdf]

Supplement E. CHW Survey
W2 Final Version: 3/18/21

Notes on how to read this survey document:

- Text in **BOLD CAPS** in brackets represents programming instructions, which will not be visible in the online survey.
- **SP** means "single punch" where only one response is allowed from the list of response options and **MP** means "multi punch" where more than one response can be selected.
- Question numbers or response list numbers will not visible in the online survey.
- The question number in (parenthesis) maps to the corresponding question in the Employer survey.

[SHOW ALL] [INTRO A]

Massachusetts Department of Public Health
2021 Community Health Worker Survey

Thank you for participating in the Community Health Worker (CHW) Survey being conducted by the Massachusetts Department of Public Health (DPH). DPH is conducting this survey to learn more about the roles of Community Health Workers and the work they do in their organizations. The information you provide will help us to support efforts to improve training and employment opportunities for Community Health Workers in Massachusetts. Your participation is greatly appreciated.

Please note the following:

- The words "your organization" in this survey refer to **[INSERT SITE NAME]**.
- The term "CHWs" is used in this survey to refer to Community Health Workers.

[ASK ALL] [SP] [REQUIRE ANSWER] [PROMPT IF LEFT BLANK: You must respond to this question in order to proceed with the survey.**]**

CA1. Our records indicate that you are a Community Health Worker (CHW). CHWs are known by many titles, including Outreach Worker, Patient Navigator, Community Health Advisor, Peer Navigator, Community Health Representative, Community Health Advocate, Promotora, Family Advocate, and many other names.

Please confirm that you are a Community Health Worker (CHW) at your organization.

1 Yes
2 No

[TERMINATE IF CA1=2 (NO)]

Thank you for your time. You are not eligible to take this survey.

[EXIT]

Please click the "OK" button to exit.

[ASK IF CA1=1 (YES)] [SP] [DROP DOWN FOR NUMBER OF YEARS: Less than 1 year, 1 year, 2 years, 3 years …. 40 years, More than 40 years]

CA2. For how many years have you been working as a Community Health Worker (CHW) in the United States?

DROPDOWN "Number of Years"

[SHOW IF CA1=1 (YES)] [INTRO B]

Your Employment as a CHW

The next few questions are about your employment as a Community Health Worker (CHW) at your organization. As a reminder, by "your organization" we are referring to **[INSERT SITE NAME]**.

[ASK IF CA1=1 (YES)] [SP] [PROMPT IF LEFT BLANK: We would really like to have your response to this question.**]**

CB1. Which one of the following best describes your employment as a CHW at your organization? *Please select one response.*

1 Full-time (at least 30 hours per week), paid
2 Part-time (less than 30 hours per week), paid
3 Volunteer, unpaid

[ASK IF ORGANIZATION_TYPE= CBO and CA1=1 (YES)] [MP]

CB2. (EA2) Which one of the following best describes the type of organization you work for as a CHW? *If you work for more than one type of organization, please select the one you work for the most.*

1 Community Action Agency
2 Disability Services
3 Domestic Violence Services
4 Elder Services
5 HIV/AIDS Services
6 Housing Support
7 Rape Crisis
8 Legal Services
9 Refugee/Immigrant
10 Substance Use Services
11 Other Mental/Behavioral Health Services
12 Teen Health
13 Veteran Services
14 Other (Please specify ________________________)

[ASK IF CA1=1 (YES)] [SP]

CB3. Which of the following **is closest to** your job title? *Please select one response.*

1 Case Manager
2 Care Coordinator
3 Community Care Coordinator
4 Community Health Advocate
5 Community Health Educator
6 Community Health Representative
7 Community Health Worker
8 Community Navigator
9 Doula
10 Home Visitor/Support Worker
11 Lactation Consultant/Specialist
12 Outreach Counselor
13 Outreach Specialist
14 Outreach Worker
15 Patient Advocate
16 Patient Navigator
17 Peer Counselor
18 Peer/Teen Educator
19 Promotora
20 Public Health Aide
21 Other (Please specify ___________________)

[ASK IF CA1=1 (YES)] [NUMERIC BOX, RANGE = 0 TO 12] [PROMPT IF LEFT BLANK: We would really like to have your response to this question**. IF RESPONSE EXCEEDS RANGE, PROMPT:** You have entered a very large number; please double-check your response**]**

CB4. In the past 12 months, how many months in total did you work as a CHW at your organization? *Please provide your best estimate.*

____________ months

[ASK IF CA1=1 (YES)] [NUMERIC BOX, RANGE = 1 TO 99. IF "0" PROMPT: You indicated that you are currently employed as a community health worker. Please adjust your answer**. IF RESPONSE EXCEEDS RANGE, PROMPT:** You have entered a very large number of hours; please double-check your response**]**

CB5. How many hours per week do you work on average as a CHW at your organization?

____________ hours per week

[ASK IF CB1=1 OR 2 (FULL TIME OR PART TIME)] [NUMERIC BOX, RANGE = $0 TO $80,000. IF "0" PROMPT: You indicated that you are currently employed as a Community Health

Worker. Please adjust your answer**. IF RESPONSE EXCEEDS RANGE, PROMPT:** You have entered a very large number; please double-check your response**]**

CB6. (ED3) What is your income per year as a CHW at your organization? *Please provide your best estimate.*

____

[ASK IF CB1=1 OR 2 (FULL TIME OR PART TIME)] [SP]

CB7. (ED4) Over the past 12 months, has your income as a CHW at your organization increased, decreased, or stayed the same?

1 Increased

2 Decreased

3 Stayed the same

[ASK IF CA1=1 (YES)] [MP]

CB8. (ED5) Do you receive any of the following from your organization? *Please select all that apply. If none of these are provided, please select "none of the above."*

1 Child care

2 Commuter subsidy

3 Unpaid leave for training/education

4 Paid leave for training/education

5 Health/dental insurance

6 Mileage/parking reimbursement

7 Paid time off (vacation, sick time, personal time, etc.)

8 Pension or retirement plan

9 Tuition assistance

10 Wage/salary increase

11 Other benefit (Please specify _______________)

12 *None of the above* [SP]

[SHOW IF CA1=1 (YES)] [INTRO C]

Your Role as a Community Health Worker (CHW)

The next few questions are about your responsibilities and activities as a Community Health Worker (CHW) at your organization. As a reminder, by "your organization" we are referring to **[INSERT SITE NAME]**.

[ASK IF CA1=1 (YES)] [MP] [PROMPT IF LEFT BLANK: We would really like to have your response to this question.]

CC1. (EC1) Is your work as a CHW at your organization related to any of the following **specific health issues or chronic diseases**? *Please select all that apply.*

1 Asthma

2 Cancer
3 Cholesterol management
4 COVID-19
5 Dementia/Alzheimer's
6 Diabetes
7 Disabilities (e.g., physical, developmental)
8 Falls prevention
9 Heart disease
10 Hepatitis (Hep B, Hep C)
11 HIV/AIDS
12 Hypertension
13 Maternal and infant health
14 Obesity
15 Oral health
16 Sexual health
17 Substance use
18 Other Mental/Behavioral health
19 Tobacco cessation
20 Tuberculosis
21 Other health issue/chronic disease (Please specify ____________)
22 *None of the above* [SP]

[ASK IF CA1=1 (YES)] [RANDOMIZE LIST; ANCHOR ITEMS 17 AND 18 LAST] [MP]
[PROMPT IF LEFT BLANK: We would really like to have your response to this question.]
CC2. (EC3) Which of the following **health promotion and disease prevention related activities** do you perform as a CHW at your organization? *Please select all that apply.*

1 Care coordination (e.g., referrals to medical or community-based services)
2 Case management
3 Chronic disease self-management (e.g., Diabetes Prevention Program, My Life My Health, etc.)
4 Communicating between providers and patients/families
5 Contact tracing for COVID-19
6 Other COVID-19 related activities
7 Teaching health literacy
8 Individual or community needs assessment
9 Individual or community outreach and education
10 Informal support/counseling
11 Medical appointments (e.g., scheduling, maintaining, etc.)
12 Helping patients with medication/treatment adherence

13 Motivational interviewing
14 Promoting healthy lifestyles (e.g., nutrition, exercise, etc.)
15 Support groups (e.g., organizing, leading)
16 Assisting with transitions of care (e.g., post hospital discharge)
17 Other health promotion activity (Please specify _______________)
18 *None of the above* [SP]

[ASK IF CC2=3 (CHRONIC DISEASE SELF-MANAGEMENT)] [MP]

CC3. (EC5) Do you link or refer individuals to/from any of the following **chronic disease management community resources**? *Please select all that apply.*

1 Diabetes Prevention Program (DPP)
2 Diabetes Self-Management Education Program (DSME)
3 Chronic Disease Self-Management Education Program (CDSME)/My Life My Health
4 Blood Pressure Self-Management Program
5 Other chronic disease management community resource (Please specify____________)
6 *None of the above* [SP]

[ASK IF CA1=1 (YES)] [MP]

CC4. (EC6) Which of the following **services** do you connect clients or patients to? *Please select all that apply.*

1 Child care
2 Clothing and other household goods
3 Education assistance/resources
4 Employment
5 Food security
6 Fuel/utility assistance
7 Health insurance enrollment
8 Housing
9 Income assistance
10 Legal services
11 Long-term services and supports (e.g., home care, adult day programs)
12 Translation/interpretation
13 Transportation
14 Violence prevention (e.g., shelter)
15 Payment assistance for medication
16 Support with mental/ behavioral health
17 Other non-medical service (Please specify ____________________)
18 *None of the above* [SP]

[ASK IF CA1=1 (YES)] [GRID: SP ACROSS, MP DOWN]

CC4a. (EC6a) Thinking of all the work you perform as a CHW for your organization, how much of your time do you spend at each of the following locations? *Please select a response for each item.*

	None of your time 1	Some of your time 2	Most of your time 3
a. Your organization's location			
b. At another organization's location			
c. In the community			

[ASK IF CA1=1 (YES)] [MP]

CC5. (EC7) Which of the following **special populations** do you work with? *Please select all that apply.*

1 Children/adolescents
2 Farm workers
3 Foreign nationals/immigrants/refugees
4 History of frequent Emergency Department (ED) use
5 History of frequent hospitalization
6 Homeless individuals
7 Individuals without a Primary Care Provider
8 Isolated rural residents
9 Seniors (ages 65 and up)
10 Sexual minorities (i.e., LGBTQ individuals)
11 Pregnant women and infants
12 Uninsured individuals
13 Other special populations (Please specify ____________________)
14 *None of the above* **[SP]**

[ASK IF CA1=1 (YES)] [SP]

CC6. (EC8) Do you mostly work with **specific race/ethnicity groups**?

1 Yes
2 No

[ASK IF CC6=1(YES)] [MP]

CC7. (EC9) Which **specific race/ethnicity groups** do you mostly work with? *Please select all that apply.*

1 Black or African American
2 Hispanic or Latino
3 Asian
4 American Indian or Alaska Native

5 Native Hawaiian or Other Pacific Islander
6 White
7 Other race/ethnic group (Please specify ________________)

[ASK IF CA1=1 (YES)] [SP]
CC8. (EC10) Does your organization use patient electronic health records (EHRs)?

1 Yes
2 No
3 Don't know

[ASK IF CC8=1 (YES)] [SP]
CC9. (EC11) Do you have access to patients' electronic health records (EHRs) at your organization?

1 Yes
2 No

[ASK IF CC9=1 (YES)] [MP]
CC9a. (EC11a) Which of the following are you able to do with patients' electronic health records (EHRs) at your organization? *Please select all that apply.*

1 Read the patient registry list
2 Enter notes into a patient record
3 Look up information on the patient record (e.g., needed/completed tests, appointments kept/missed, etc.)
4 Schedule appointments
5 Other (Please specify ________________)

[ASK IF CC8=1 (YES)] [SP]
CC10. (EC12) Are the services/activities you provide as a CHW included in patients' electronic health records (EHRs)?

1 Yes
2 No

[ASK IF CA1=1 (YES)] [SP]
CC11. (EC13) Do you receive supervision at your organization in the work you do as a CHW?

1 Yes
2 No

[ASK IF CC11=1 (YES)] [SP]
CC12. Who supervises you at your organization?

1 Clinical/Medical Director
2 Executive Director

3 Practice Manager
4 Program Manager/Director
5 Project Coordinator
6 Project Director
7 Team Leader
8 Volunteer Coordinator
9 Community Health Worker (CHW)
10 Senior CHW
11 Case Manager
12 Nurse Manager
13 Social Worker
14 Behavioral health care provider (e.g., psychiatrist, mental health counselor, etc.)
15 Other (Please specify_________________)

[ASK IF ORGANIZATION_TYPE = HOSPITAL OR CHC AND CA1=1 (YES)] [SP] [PROMPT IF LEFT BLANK: We would really like to have your response to this question.**]**

CC13a. (EC16a) Do you **work directly with members of a clinical care team** (i.e., nurses, social workers, primary care providers, mental/behavioral health providers)?

1 Yes
2 No

[ASK IF CC13a=1 (YES)] [MP] [PROMPT IF LEFT BLANK: We would really like to have your response to this question.**]**

CC13b. (EC16b) Which of the following types of members of a clinical care team do you **work directly with** at your organization? *Please select all that apply.*

1 Behavioral health care provider (e.g., psychiatrist, mental health counselor, etc.)
2 Primary care provider (physician, nurse practitioner, physician's assistant)
3 Specialist (e.g., oncologist, cardiologist, OB-GYN, etc.)
4 Registered nurse
5 Social worker
6 Other clinical providers (Please specify _______________)

[ASK IF CC13a=1 (YES)] [MP]

CC14a. (EC17a) In which of the following ways do you **work directly with members of a clinical care team**? *Please select all that apply.*

1 Participate in regular meetings
2 Meet on an as-needed basis
3 Receive patient or client referrals
4 Help develop care plans
5 Help implement care plans

6 Participate in case reviews

7 **[SHOW IF CC9=1 (YES)]** Document patient encounters in the same electronic health record (EHR) as other members of the clinical care team

8 Other (Please specify ________________)

[ASK IF CA1=1 (YES)] [SP]

CC14b. (EC17b) Do you communicate about patients or clients regularly with administrative staff?

1 Yes

2 No

[ASK IF CA1=1 (YES)] [GRID: SP ACROSS, MP DOWN]

CC15. (EC18) Thinking of the organization you work for as a CHW, please indicate how much you agree or disagree with each of the following statements.

	Agree completely 4	Agree somewhat 3	Disagree somewhat 2	Disagree completely 1
a. My organization values the work that I do as a CHW				
b. **[IF CC11 = 1 (YES)]** My supervisor understands the work I do				
c. The teams I work with understand the work I do				
d. I am a valued member of the teams I work with				
e. I have opportunities for promotion at my organization				

[ASK IF CA1=1 (YES)] [SP]

CC16. Do you currently work for pay in a position other than as a CHW?

1 Yes

2 No

[ASK IF CC16=1 (YES)] [MP]

CC17. Why do you work in a position other than a CHW? *Please select all that apply.*

1 Community health work is not my primary occupation

2 I work at another job to supplement my income as a CHW

3 I volunteer as a CHW

4 Other (Please specify__________________)

[SHOW IF CA1=1 (YES)] [INTRO D]

CHW Training

The next few questions are about the education or training that you have as a Community Health Worker.

[ASK IF CA1=1 (YES)] [MP]

CD1. (EE5) Which of the following types of training do you have? *Please select all that apply.*

1 80 hours of CHW core competency training
2 Chronic disease self-management (e.g., Diabetes Prevention Program, My Life My Health, etc.)
3 Motivational interviewing
4 Medical interpreting training
5 Patient navigation/Navigator training
6 Emergency response training (e.g., first aid, CPR, etc.)
7 Other CHW-specific training (Please specify _______________)
8 *None of the above* [SP]

[ASK IF CD1=1 (CHW CORE COMPETENCY TRAINING)] [MP]

CD2. From which of the following sources have you received 80 hours of CHW core-competency training? *Please select all that apply.*

1 Community Health Education Center (CHEC), Boston
2 Community Health Education Center (CHEC), Lowell
3 DPH Patient Navigator Program
4 Center for Health Impact (formerly Outreach Worker Training Institute), Worcester
5 Holyoke Community College
6 Western Massachusetts Public Health Training Center
7 MassBay Community College
8 Bunker Hill Community College
9 Berkshire Community College
10 Greenfield Community College
11 Northern Essex Community College
12 North Shore Community College
13 Justice Resource Institute
14 Cambridge Health Alliance
15 At your organization
16 Other (Please specify________________)

[ASK IF CA1=1 (YES)] [MP]

CD3. (EE4) Which of the following **specific health issues or chronic diseases** do you have training in? *Please select all that apply.*

1 Asthma
2 Cancer

3 Cholesterol management
4 COVID-19
5 Dementia/Alzheimer's
6 Diabetes
7 Disabilities (e.g., physical, developmental)
8 Falls prevention
9 Heart disease
10 Hepatitis (Hep B, Hep C)
11 HIV/AIDS
12 Hypertension
13 Maternal and infant health
14 Obesity
15 Oral health
16 Sexual health
17 Substance use
18 Other Mental/Behavioral health
19 Tobacco cessation
20 Tuberculosis
21 Other health issue/chronic disease (Please specify ____________)
22 *None of the above* [SP]

[ASK IF CA1=1 (YES)] [GRID: SP ACROSS, MP DOWN]

CD4. For each area listed below, please indicate the extent to which you are interested in being trained or receiving additional training in that area.

	Very interested 3	Somewhat interested 2	Not at all interested 1
a. Asthma			
b. Cancer			
c. Cholesterol management			
d. COVID-19			
e. Dementia/Alzheimer's			
f. Diabetes			
g. Disabilities (e.g., physical, developmental)			
h. Falls prevention			
i. Heart disease			
j. Hepatitis (Hep B, Hep C)			
k. HIV/AIDS			

l. Hypertension			
m. Maternal and infant health			
n. Obesity			
o. Oral health			
p. Sexual health			
q. Substance use			
r. Other Mental/Behavioral health			
s. Tobacco cessation			
t. Tuberculosis			
u. Other training (Please specify ____________)			

[ASK IF CA1=1 (YES)] [GRID: SP ACROSS, MP DOWN]

CD4a. (EE6a) Have you ever received health and safety training on the following topics at your organization?

	Yes<br>1	No<br>2	Not sure<br>3
a. Hazards on the job			
b. Workers' rights under Occupational Safety and Health Administration (OSHA)			
c. Workers' Compensation			

[ASK IF CA1=1 (YES)] [SP] [PROMPT IF LEFT BLANK: We would really like to have your response to this question.]

CD5. (EE7) Before today, were you aware that there is a CHW Board of Certification in the state of Massachusetts?

1 Yes
2 No
3 Not sure

[ASK IF CD5=1 (YES)] [SP]

CD6. (EE8) Are you certified by the Massachusetts CHW Board of Certification?

1 Yes
2 No

[ASK IF CD6=1 (YES)] [DROP DOWN FOR MONTHS – JANUARY THROUGH DECEMBER] [DROP DOWN FOR YEARS – SHOW "2015 or earlier" FOLLOWED BY EACH YEAR UP TO CURRENT YEAR]

CD7. When did you first become certified by the Massachusetts CHW Board of Certification?

[DROPDOWNS FOR MONTH "Please select month" AND YEAR "Please select year"]

[ASK IF CD6=1 (YES)] [SP]

CD8. Now please think about what you had to do to get certified by the Massachusetts CHW Board of Certification. How would you rate the process of getting certified?

1 Very easy
2 Somewhat easy
3 Somewhat difficult
4 Very difficult

[ASK IF CD6=1 (YES)] [SP]

CD9. How likely are you to maintain or renew your CHW Board Certification?

1 Very likely
2 Somewhat likely
3 Somewhat unlikely
4 Very unlikely

[ASK IF CD9=3 OR 4 (SOMEWHAT UNLIKELY OR VERY UNLIKELY)] [ALLOW UP TO 3 ITEMS TO BE SELECTED] [PROMPT IF MORE THAN 3 ITEMS ARE SELECTED: "Please select up to 3 items.**"] [MP]**

CD10. Please select the top 3 reasons why you are ***[INSERT RESPONSE FROM CD9]*** to maintain or renew your CHW Board Certification.

1 There are too many requirements to maintain certification
2 I do not have the time to maintain the certification
3 I do not have the resources to pay for renewing the certification
4 Not enough instructors to provide continuing education for recertification
5 Not enough job opportunities to make renewing certification worthwhile
6 I don't see any benefits to maintaining certification
7 Training is not offered in my language of choice
8 Other (Please specify ____________)

[SHOW IF CA1=1 (YES)] [INTRO D1]

As you may know, a Massachusetts CHW Board of Certification was established at the Department of Public Health in 2012 to develop a process for voluntary certification of CHWs. The purpose is to better define CHWs' work, establish training standards, and promote job creation and stable funding for CHWs. Certification became available in October 2018 (experience pathway only).

[ASK IF (CD6 DOES NOT EQUAL 1 (YES)] [SP]

CD11. How interested are you in being a Board-Certified CHW?

1 Very interested
2 Somewhat interested
3 Somewhat uninterested
4 Very uninterested

[ASK IF CD11=3 OR 4 (SOMEWHAT UNINTERESTED OR VERY UNINTERESTED)] [ALLOW UP TO 3 ITEMS TO BE SELECTED] [PROMPT IF MORE THAN 3 ITEMS ARE SELECTED: "Please select up to 3 items."] [MP]

CD12. Please select the top 3 reasons why you are ***[INSERT RESPONSE FROM CD11]*** in being a Board-Certified CHW.

1 I don't know enough about it
2 There are too many requirements to obtain certification
3 I don't think I meet the requirements
4 I do not have the resources to pay for the certification
5 Not enough job opportunities to make certification worthwhile
6 I don't see any benefits to getting certified
7 Training is not offered in my language of choice
8 Other (Please specify ____________)

[ASK IF CA1=1 (YES)] [GRID: SP ACROSS, MP DOWN]

CD13. (EE10) Below are some statements regarding the **value of CHW certification to you as a CHW**. Please indicate the extent to which you agree or disagree with each statement.

CHW certification will help you…	Agree completely 4	Agree somewhat 3	Disagree somewhat 2	Disagree completely 1
a. (EE10_a) Better define your role as a CHW				
b. (EE10_b) Learn new skills				
c. (EE10_c) Improve the work you do as a CHW				
d. (EE10_d) Take on increased responsibilities in your work				
e. (EE10_e) Win more respect from the individuals you serve				
f. (EE10_f) Win more respect from other professionals				
g. (EE10_g) Better integrate with other teams				
h. (EE10_h) Increase your job security				
i. (EE10_j) Get promoted in your job as a CHW				
j. Increase your salary as a CHW				
k. Improve your job opportunities				
l. Increase your self-confidence as a CHW				
m. Increase your employer's confidence in your abilities				

[ASK IF CA1=1 (YES)] [SP]
CD14. (EE2_2) Does your employer require CHW Board Certification?

1 Yes
2 No
3 Not sure

[SHOW IF CA1=1 (YES)] [INTRO E]
About You

The last few questions are about you.

[ASK IF CA1=1 (YES)] [SP]
CE1. What is your age now?

1 18 to 24
2 25 to 34
3 35 to 44
4 45 to 54
5 55 to 64
6 65 or older

[ASK IF CA1=1 (YES)] [SP]
CE2. What is your gender?

1 Male
2 Female
3 Transgender
4 Other

[ASK IF CA1=1 (YES)] [SP]
CE3. What is the highest grade or level of school that you have completed?

1 8th grade or less
2 Some high school, but did not graduate
3 High school graduate or GED
4 Some college or 2-year degree
5 4-year college graduate
6 More than 4-year college degree

[ASK IF CA1=1 (YES)] [SP]
CE4. Are you of Hispanic or Latino origin or descent?

1 Yes, Hispanic or Latino
2 No, Not Hispanic or Latino

[ASK IF CA1=1 (YES)] [MP]

CE5. What is your race? *(Check all that apply)*

1 White

2 Black or African American

3 Asian

4 Native Hawaiian or Other Pacific Islander

5 American Indian or Alaska Native

6 Other (Please specify ____________)

[ASK IF CA1=1 (YES)] [MP]

CE6. In which of the following languages are you fluent enough to communicate with the individuals you serve as a CHW if you do not use an interpreter? *(Check all that apply)*

1 English

2 American Sign Language (ASL)

3 Arabic

4 Cambodian/Khmer

5 Chinese (includes Mandarin, Cantonese)

6 Haitian/Creole

7 Laotian

8 Portuguese

9 Russian

10 Spanish

11 Vietnamese

12 Other (Please specify ____________)

[THANK AND CLOSE]

Thank you. Please click the "OK" button to submit your responses.
