# Supplementary material for: Evaluating the impact of community health worker certification in Massachusetts: Design, methods, and anticipated results of the Massachusetts community health worker workforce survey
Source: Front Public Health. 2023 Jan 12;10:1043668. doi: 10.3389/fpubh.2022.1043668 (PMC9877511; doi:10.3389/fpubh.2022.1043668)
Supplement: Supplementary file 6 [file Data_Sheet_5.pdf]

Supplement F. CHW Fact Sheet for Interviewers
Interviewer Fact Sheet for Contact and Reminder Calls
Final Version: July 21, 2020

Background:
The Massachusetts Department of Public Health (DPH) is requesting contact information for Community Health Workers (CHWs) and their supervisors to conduct an important survey in the next few months about the role and work of CHWs. DPH will use the survey results to better support funding, training, and employment for CHWs in Massachusetts.

Frequently Asked Questions:

What is the survey about?
The survey asks about the role and work of CHWs in your organization.

Why should I provide contact information for the survey?
DPH will use the survey results to better support funding, training, and employment for CHWs in Massachusetts.

How will the contact information be used?
We will use the information to contact CHWs and their supervisors about the survey in the next few months. The information you provide will be kept confidential.

What is a Community Health Worker (CHW)?
"Community Health Worker" or CHW is an umbrella term for a number of job titles that perform one or more of the CHW roles. CHWs work in a variety of settings and are distinguished from other public health professionals because they are hired primarily for their special connection to and understanding of the populations and communities they serve. Some of the most common job titles for CHWs in Massachusetts are:

- Community Health Educator
- Enrollment Worker
- Family Advocate
- Family Planning Counselor
- Doula
- Family Support Worker
- Health Advocate
- Health Educator
- HIV Peer Advocate
- Outreach Worker
- Outreach Educator
- Patient Navigator
- Peer Advocate
- Peer Leader
- Promotor(a)
- Promotor(a) de Salud
- Street Outreach Worker

Who is collecting the contact information?

The Office of Survey Research (OSR) of the University of Massachusetts Chan Medical School is collecting contact information for the upcoming survey on behalf of DPH. If you have any additional questions about this information request, you can call the Office of Survey Research at this toll-free number: 1-888-368-7157.
